# Executive Summary of the American Radium Society Appropriate Use Criteria for the Management of Peritoneal Carcinomatosis From Different Tumor Origins: Systematic Review and Guidelines

**DOI:** 10.1002/cam4.71043

**Published:** 2025-07-22

**Authors:** Expert Panel Management of Peritoneal Carcinomatosis, Timothy Kennedy, Sareena Singh, Gerard Abood, Eric Christenson, Christopher J. Anker, Dmitriy Akselrod, Christopher L. Hallemeier, Krishan R. Jethwa, Ed Kim, Percy Lee, Eric D. Miller, Neil B. Newman, J. Eva Selfridge, Navesh Sharma, William Small, Leila Tchelebi, Vonetta M. Williams, Suzanne Russo

**Affiliations:** ^1^ Department of Surgery Rutgers Cancer Institute New Brunswick New Jersey USA; ^2^ Department of Gynecologic Oncology MetroHealth Cleveland Ohio USA; ^3^ Department of Surgery Loyola University Stritch School of Medicine Maywood Illinois USA; ^4^ Division of Medical Oncology, Department of Medicine Johns Hopkins University Baltimore Maryland USA; ^5^ Division of Radiation Oncology University of Vermont Larner College of Medicine Burlington Vermont USA; ^6^ Department of Radiology University of Vermont Larner College of Medicine Burlington Vermont USA; ^7^ Department of Radiation Oncology Mayo Clinic College of Medicine Rochester Minnesota USA; ^8^ Department of Radiation Oncology University of Washington Seattle WA USA; ^9^ Department of Radiation Oncology City of Hope National Medical Center Los Angeles California USA; ^10^ Department of Radiation Oncology, James Cancer Center The Ohio State University Columbus Ohio USA; ^11^ Department of Radiation Oncology University of Texas San Antonio Health Science Center San Antonio Texas USA; ^12^ Department of Medical Oncology University Hospitals, Case Western Reserve University School of Medicine Cleveland Ohio USA; ^13^ Department of Radiation Oncology WellSpan Cancer Center York Pennsylvania USA; ^14^ Department of Radiation Oncology, Cardinal Bernadin Cancer Center Loyola University Stritch School of Medicine Maywood Illinois USA; ^15^ Department of Radiation Oncology Donald and Barbara Zucker School of Medicine Hofstra/Northwell Hempstead New York USA; ^16^ Department of Radiation Oncology Memorial Sloan Kettering New York New York USA; ^17^ Department of Radiation Oncology MetroHealth, Case Western Reserve University School of Medicine Cleveland Ohio USA

**Keywords:** cytoreductive surgery, early postoperative intraperitoneal chemotherapy (EPIC), hyperthermic intraperitoneal chemotherapy (HIPEC), intraperitoneal, peritoneal carcinomatosis, pressurized intraperitoneal aerosolized chemotherapy (PIPAC)

## Abstract

**Background:**

Multimodality therapy incorporating a combination of cytoreductive surgery (CRS), intraperitoneal (IP) and systemic therapy continues to evolve for peritoneal carcinomatosis (PC) However, treatment and outcomes vary depending on tumor of origin.

**Aims:**

To develop Appropriate Use Criteria (AUC) guidelines to facilitate treatment decision‐making for patients with PC based on available evidence.

**Materials and Methods:**

The American Radium Society (ARS) multidisciplinary expert panel performed a comprehensive systematic review. Preferred Reporting Items for Systematic Reviews and Meta‐analyses (PRISMA) methodology was used. These studies were used to inform the expert panel, which then rated the appropriateness of various treatments in seven representative clinical scenarios through a well‐established modified Delphi consensus methodology.

**Results:**

Treatment of PC is often treated with a combination of CRS and IP ± systemic chemotherapy but specific recommendations exist for different tumor types and outcomes vary.

**Discussion:**

Treatment of PC is complex and varies depending on origin of primary tumor and extent of disease. These AUC assist in patient and treatment selection for different clinical scenarios.

**Conclusion:**

A summary of recommendations is outlined to guide practitioners on the management of PC from different tumor origins.

## 1 Introduction

Peritoneal carcinomatosis (PC) is a heterogeneous disease involving malignant tumor cell deposits in the serous membrane lining of the peritoneum. PC can arise as a primary peritoneal malignancy, from local tumor invasion, or distant metastases, and usually presents as advanced disease associated with poor prognosis. The incidence of PC varies based on the tumor of origin [1]. Peritoneal metastasis is the most common cause of PC. Peritoneal involvement is found at the time of diagnosis in up to 75% of ovarian cancer [2], 14% of gastric cancer [3], 18% of pancreatic cancer [4], and 5%–10% of colorectal cancer (CRC) cases [5]. Other less common abdominopelvic malignancies that have been known to metastasize to the peritoneum include appendiceal, biliary tract, liver, and genitourinary tract cancers [6]. PC caused by extra‐abdominopelvic malignancy metastasis occurs less frequently and is estimated to represent 9% of all PC cases [7] and occur at frequencies that vary according to primary tumor stage, bulk of disease, and histology [6]. Breast (41%), lung (26%), and melanoma (9%) are most common [6].

The most common histologies associated with PC are serous, mucinous, signet ring, or clear cell and depend on the organ of origin [6]. Serous carcinoma accounts for approximately 60% of all PC cases and is typically associated with advanced ovarian or fallopian tube cancer but can be diagnosed as primary peritoneal carcinoma (PPC) in 10%–15% when primary cancer is not identified [6]. Subtypes of PPC include extraovarian primary peritoneal carcinoma (EOPPC), serous surface carcinoma, serous carcinoma of the peritoneum, and extra ovarian Mullerian adenocarcinoma [8]. Other subtypes of PPC include leiomyosarcomas, leiomyomatosis peritonealis disseminata, and primary peritoneal mesothelioma [9]. Primary peritoneal mesothelioma is caused by asbestos exposure in 33%–50% of cases and occurs mostly in older males (60 years and older) [10].

Advancements in the understanding of tumor biology and pathways of intraperitoneal (IP) spread have led to the concept of PC being a locoregional disease (in the absence of systemic metastases) for some disease entities. Multiple studies have suggested a potential survival benefit to multimodal therapy compared with traditional palliative approaches (60 vs. 4–12 months, respectively) [11]. Multimodal therapy incorporating a combination of cytoreductive surgery (CRS), IP, and systemic therapy continues to evolve for PC, to improve locoregional control and survival rates in carefully selected patients.

The present comprehensive systematic review and guidelines is intended to facilitate treatment decision making for patients with PC, based on the available evidence. The Population, Intervention, Comparator, Outcome, Timing, and Study Design (PICOTS) questions included (1) What is the impact of histology and molecular profile on selection of therapies and outcomes for patients with PC?, (2) What are the optimal treatment strategies for patients with PC from gynecologic malignancies?, (3) What are the optimal treatment strategies for patients with PC from gastrointestinal malignancies?, (4) What are the optimal treatment strategies for patients with PC from other histologies?, and (5) What are palliative treatment options for patients who are unsuitable or decline CRS ± IP therapy in patients with PC? Herein we describe the data in support of various treatment options and provide American Radium Society (ARS) appropriate use criteria (AUC) for the treatment of PC.

## 2 Methodology

**FIGURE 1:**
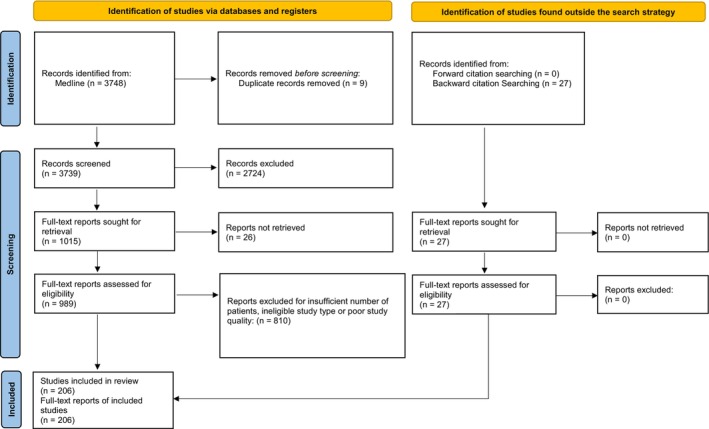
PRISMA 2020 [12, 13] study selection flow diagram for management of peritoneal carcinomatosis from different tumor origins.

This ARS AUC surgical specialty‐led committee is comprised of all key specialties (gynecologic oncology, surgical oncology, colorectal surgery, medical oncology, radiation oncology, and radiology). Using the Population, Intervention, Comparator, Outcome, Timing and Study Design (PICOTS) framework, the evidence regarding treatment outcomes was assessed using Cochrane and PRISMA 2020 methodology [12, 13]. Eligible studies included prospective Phase II–III trials, meta‐analyses, and retrospective analyses published between 1/1/2004 and 2/23/2024 in the Ovid Medline database. Trial size required for inclusion was > 25 patients. Table 1 contains the database search strategy designed by a librarian‐trained information specialist. Two authors independently screened the comprehensive list of articles, and one assessed the full text articles to determine the final studies included in the Summary of the Literature Review which advised our recommendations (Figure 1). Discrepancies between the reviewers were resolved by consensus. Of the 989 articles identified using the search strategy, 179 were selected for inclusion that met all inclusion criteria. Twenty‐seven additional studies were included through backward citation searching that significantly contributed to the literature were identified from the reference list of articles found from the search strategy. No studies identified by forward citation searching were included. Study type and quality for these references were assessed via ARS AUC methodology using the systematic review PRISMA 2020 checklist confirming completion of essential elements. Well‐established RAND‐UCLA consensus methodology (modified Delphi) was used by the expert panel to rate the appropriateness of the treatment options [14], with a total of three rounds of voting employed by the group. Categories included (1) usually not appropriate (U, score 1–3); (2) may be appropriate (M, score 4–6); and (3) usually appropriate (A, score 7–9). An additional 21 studies within the introduction and future directions sections are referenced only to provide context but are not included as the supporting evidence for oncologic interventions unless they are also cited within the “Summary of Literature Review” as they were not used by the committee to guide recommendations. In sum the authors added 48 citations from bibliographies, websites, or books not found in the literature search, resulting in 227 references total selected for inclusion. Of the 206 references used as evidence, 206 are categorized as therapeutic references including 38 well‐designed studies (Phase II randomized and Phase III), 31 moderately well‐ designed studies that account for most common biases (matched cohort and Phase II studies), 128 studies with design limitations (retrospective reviews), 4 supplemental studies (not useful as primary evidence), and 5 references that are meta‐analysis studies. The project proposal as well as this executive summary were reviewed and approved by the ARS AUC steering committee, which includes a librarian with expertise in systematic reviews. For further details on ARS AUC methodology guidelines. See https://www.americanradiumsociety.org/page/aucmethodology.

**TABLE 1:** Management of peritoneal carcinomatosis of different tumor origins search strategy.

Performed on February 23, 2024
Database: Ovid MEDLINE(R) All Search strategy: **1** (“1938510” or “24614244” or “24269290” or “20363414” or “20363414” or “23680178” or “20541198” or “20844363”).ui. (7) **2** (“28829911” or “15800321” or “17401004” or “22646630” or “19465425” or “27429017” or “34191461” or “32024132” or “34631736” or “30089078”).ui. (10) **3** (“27859977” or “28315293”).ui. (2) **4** (“1584260” or “10235156” or “27745985” or “31655197” or “30877837” or “18164844” or “27859977” or “28623448” or “36632597”).ui. (9) **5** (“10526266” or “17185082”).ui. (2) **6** (“10680872” or “38249918” or “28258892” or “28375481” or “37149827” or “31655197” or “22631652”).ui. (7) **7** (“15069706” or “16298001” or “25175056” or “22417808” or “25489937” or “31299242” or “32160096” or “28258892” or “25344056” or “37641149”).ui. (10) **8** (“34191461” or “38182849” or “38099991”).ui. (2) **9** Esophageal Neoplasms/(60698) **10** Esophagus/(45059) **11** Neoplasms/(520661) **12** 10 and 11 (360) **13** (esophag* or oesophag* or gastroesophag* or gastro‐esophag* or gastrooesophag*).ti,ab,kf. (220517) **14** (cancer* or carcinoma* or neoplas* or squamous* or malignan* or tumor* or tumour*).ti,ab,kf. (4208787) **15** 13 and 14 (91410) **16** 9 or 12 or 15 (101910) **17** (T1a or superficial* or mucosal* or earl*).ti,ab,kf. (2466874) **18** (cervical* or neck*).ti,ab,kf. (515500) **19** limit 18 to yr=“2013‐Current” (242045) **20** ((Tracheoesophageal* or tracheo‐esophageal* or malignan*) and fistula*).ti,ab,kf. (8291) **21** stent*.ti,ab,kf. (127905) **22** *Radiotherapy/or *radiotherapy, conformal/or *radiotherapy, intensity‐modulated/or *radiotherapy dosage/or *radiotherapy planning, computer‐assisted/(57135) **23** rt.fs. (215096) **24** (radiotherap* or radiat* or irradiat* or chemoradi* or brachytherap* or IMRT or “intensity modulated radiation therapy” or VMAT or “volumetric modulated arc therapy” or proton*).ti,ab,kf. (1019511) **25** (brachytherap* or intracavitar* or interstitial* or implant*).ti,ab,kf. (638167) **26** (chemo‐radi* or chemoradi*).ti,ab,kf. or Chemoradiotherapy/(49017) **27** or/22‐26 (1672566) **28** (immunotherap* or pembrolizumab* or nivolumab* or avelumab* or durvalumab* or atezolizumab* or checkpoint inhibitor* or PD‐L1 inhibitor* or PD1 inhibitor*).ti,ab,kf. or *immunotherapy/(189580) **29** *antineoplastic agent/or *carboplatin/or *cetuximab/or *fluorouracil/or *cisplatin/or *multimodality cancer therapy/or *radiosensitizing agent/or *immunotherapy/or *monoclonal antibody/or *immunological antineoplastic agent/or *programmed death 1 receptor/or molecularly targeted therapy/or vaccine/(424727) **30** (chemotherap* or chemoradio* or carboplatin* or cisplatin* or fluorouracil* or paclitaxel* or taxol or “epidermal growth factor receptor inhibitor*” or “EGFR inhibitor*” or immunotherap* or pembrolizumab* or nivolumab* or avelumab* or durvalumab* or atezolizumab* or checkpoint inhibitor* or PD‐L1 inhibitor* or PD1 inhibitor*).ti,ab,kf. (765801) **31** or/28‐30 (1043764) **32** (CT or computed tomography or PET or postitron emission tomography or MRI or magnetic resonance imaging or endoscop*).ti,ab,kf. (1370273) **33** (biomarker* or ctDNA circulating tumor DNA or cell free DNA).ti,ab,kf. (462734) **34** 32 or 33 (1798315) **35** Watchful Waiting/(5496) **36** (“watchful wait*” or “watch and wait” or “W&W” or “watch & wait” or “active surveillance” or “expectant management” or observation* or regrowth* or “organ* preserv*”).ti,ab,kf. (1126816)
**37** (NOM or “non‐operative manage*” or “nonoperative manage*” or “nonsurgical manage” or “non‐surgical manage*”).ti,ab,kf. (12099) **38** “wait and see”.ti,ab,kf. (1861) **39** (complete clinical response* or clinical complete response*).ti,ab,kf. (2404) **40** or/35‐39 (1143142)
**41** ((cervical* or mediastin*) adj3 nod*).ti,ab,kf. (22908) **42** (neoadjuvant* or neo‐adjuvant* or preoperativ* or triple therapy or ((chemoradi* or chemoradiotherap*) and (followed by or plus) and (surger* or operati* or esophagectom*))).ti,ab,kf. (433101) **43** Neoadjuvant Therapy/(30388) **44** 42 or 43 (438169) **45** (resect* or esophagectom* or oesophagectom* or surg* or opera*).ti,ab,kf. (3576202) **46** (adjuvant* or post‐operative*).ti,ab,kf. (282291) **47** 45 or 46 (3693887) **48** (prospectiv* or phase II* or phase 2* or phase III* or phase 3* or meta‐analys* or metaanalys* or randomi* or phase IV* or phase 4*).ti,ab,kf. (1931550) **49** clinical trial, phase III/or clinical trial, phase IV/or Meta‐Analysis/or controlled clinical trial/or clinical trial/or randomized clinical trial/(786566) **50** (phase 3* or phase III* or randomi* or randoml*).ti,ab,kf. or clinical trial, phase III/or clinical trial, phase IV/or Meta‐Analysis/or controlled clinical trial/or randomized clinical trial/(1458655) **51** or/48‐50 (2615094) **52** limit 51 to yr=“1993‐Current” (2396568) **53** (((coverage or margin) adj (target or diseas* or volume*)) or gross tumo* or GTV or clinical target volume* or CTV or planning target volume* or PTV or OAR or organ* at risk or organs*‐at‐risk or elective nod* radi* or ((cervical* or mediastin*) adj3 nod*)).ti,ab,kf. (45528) **54** (((Gray or “Gray/fx” or “Gray/fraction” or “Gy/fx” or “Gray/fraction” or Gy or “Gy/f*” or Gyx*) adj3 “>“) or BED* or biologic effective dose or ablati* or ((dose adj3 (escala* or high*)) or (boost* adj3 (radiot* or radiation*)))).ti,ab,kf. (658907) **55** ((pain adj3 (relie* or decreas* or improv*)) or nausea or diarrhea or toxic* or dysphagia* or esophagitis* or oesophagitis* or ulcer or hemorrhag* or “quality of life” or “quality‐of‐life” or “patient‐reported‐outcome*” or “patient reported outcome*”).ti,ab,kf. or *radiation injuries/or *Patient Reported Outcome Measures/or Radiotherapy/ae or Radiotherapy, Intensity‐Modulated/ae or Radiotherapy, Conformal/ae (1919413) **56** (((“local*” or “locoregional*” or regional* or disease* or tumo?r) adj3 (control* or recurren*)) or surviv* or progression‐free* or recurrence‐free* or disease‐free* or metastases‐free* or ((progression* or recurrence* or disease* or metastas*) adj3 (rate* or free)) or laryngectomy* or laryn* preserv* or PFS or RFS or DFS or DMFS or response rate* or disparit*).ti,ab,kf. or Survival Rate/or Disease‐Free Survival/or Survival/or Progression‐Free Survival/or Survival Analysis/or Kaplan‐Meier Estimate/(2172176) **57** (complete response* or complete clinical response* or cCR complete pathologic* response or pathologic complete response or clinical complete response or pCR or R0 or negative margin* or pseudoprogres* or inflammat*).ti,ab,kf. (1946078) **58** or/55‐57 (5459406) **59** (Toxicit* or Quality assurance or Therapeutic ratio* or ((Dosimetr* or dose) adj5 (advantage* or benefit* or detriment* or improv* or minimi* or maximi*)) or Guideline*).ti,ab,kf. (1086942) **60** (ablat* or endoscopic mucosal resection or EMR or cryotherap* or cryoablat* or photodynamic* or PDT* or ESD* or endoscopic submucosal dissection).ti,ab,kf. (218480) **61** 1 (7) **62** 16 and 17 and (27 or 60) and 58 (2806) **63** 61 not 62 (0) **64** limit 62 to yr=“2013‐Current” (1557) **65** 2 (10) **66** 16 and (27 or 31) and 58 and 44 (5225) **67** (66 and 18) or (66 and 51) (1991) **68** 65 not 67 (0) **69** (66 and 19) or (66 and 52) (1824) **70** 3 (2) **71** 16 and (27 or 31) and 58 and 47 (9436) **72** (71 and 19) or (71 and 52) (2746) **73** 70 not 72 (0) **74** 4 (9)
**75** 16 and 27 and 58 (13003) **76** (75 and 18) or (75 and 51) (4648) **77** 74 not 76 (0) **78** (75 and 19) or (75 and 52) (3931) **79** 5 (2) **80** 16 and 20 and (21 or 27 or 31) and 58 (458)
**81** 79 not 80 (0) **82** limit 80 to yr=“2013‐Current” (182) **83** 6 (7) **84** 16 and 27 and (58 and 54) (3599) **85** 83 not 84 (0) **86** (84 and 19) or (84 and 52) (1549) **87** 83 not 86 (1) **88** 7 (10) **89** 16 and 27 and (53 or 58 or 59) and 24 (13033) **90** 88 not 89 (0) **91** (89 and 19) or (89 and 52) (3988) **92** 88 not 91 (1) **93** 64 or 69 or 72 or 78 or 82 or 86 or 91 (6188) **94** limit 93 to english language (5776) **95** limit 94 to “review articles” (833) **96** meta‐analys*.ti,ab,kf. (302523) **97** 95 and 96 (173) **98** (94 not 95) or 97 (5116) **99** limit 98 to (“newborn infant (birth to 1 month)” or “infant (1 to 23 months)” or “preschool child (2 to 5 years)” or “child (6 to 12 years)”) (18) **100** (((line* or stem*) adj3 cell*) or mice* or mouse* or equine* or murine* or animal* or in vivo* or “in vitro*” or “autophag*” or culture* or ELISA or bacilus* or drug screen or nano* or quasi‐experimental* or ligand*).ti,ab,kf. or exp Mice/(6805614) **101** limit 98 to (case reports or letter or editorial or clinical trial protocol) (299) **102** (“case report” or “case‐report” or “case‐series*” or “case series*” or “narrative review” or editorial* or letter* or “response to comment*”).ti,ab,kf. (790408) **103** (protocol* or (phase I or phase 1)).ti. (122600) **104** (SEER or NCDB or “national cancer database” or “Surveillance, Epidemiology, and End Results”).ti,ab,kf. (23315) **105** exp Markov Chains/or exp Cost‐Benefit Analysis/(106968) **106** ((quality adj3 adjusted) or (cost* adj3 effective*) or markov*).ti,ab,kf. (227691) **107** (cost* adj3 effective*).ti,ab,kf. (197348) **108** exp Palliative Care/or exp Palliative Medicine/or palliati*.ti,ab,kf. (118738) **109** (GIST or gastrointestinal stromal* or sarcoma* or melanoma* or breast* or meningioma* or tuberculos* or pancrea* or bile duct* or periampullary* or neuroendocrine* or prostat* or gallbladder* or ACTH or gastrinoma* or cholangiocarcinoma* tuberculos* or thyroid* or bladder* or renal or testicular or seminoma* or (lung adj3 (cancer or carcinoma*)) or colon* or ovar* or reirradiat* or second‐line* or second line* or reflux* or PPI or proton‐pump* or pump inhibitor or gastric‐acid* or acid* or re‐irradiat*).ti,kf. (3174753) **110** 98 not (or/99‐109) (3191) **111** limit 110 to yr=“2013‐Current” (3130)

## 3 General Treatment Considerations

The optimal therapeutic approach for PC uses a multimodal treatment strategy that may vary depending on tumor histology, molecular profile, and patient factors such as performance status. Aggressive treatment strategies typically combine CRS with or without IP therapy, and consideration of neoadjuvant or adjuvant therapies. Chemotherapy, targeted therapy, and/or immunotherapy may be options depending on tumor type of origin and molecular profile.

### 3.1 Peritoneal Carcinomatosis Index

The peritoneal cancer index (PCI) is a measure of disease spread developed for PC that can be evaluated surgically (laparotomy/laparoscopy) or radiographically (CT/MRI). The PCI assesses the spread of tumors in 13 abdominal regions, each of which gets a score of 0–3. The PCI scoring system assesses tumor size accordingly: (A) 0 points for the absence of identifiable disease; (B) 1 point for lesions 0.5 cm or smaller; (C) 2 points for lesions bigger than 0.5 cm but smaller than 5 cm; and (D) 3 points for lesions or tumor mass 5 cm or bigger. The total score ranges from 0 to 39, with higher PCI scores associated with worse prognosis and can assist in patient selection with specific tumor types (such as CRC), but not for all tumor types (such as pseudomyxoma peritonei [PMP]). The PCI can also predict the outcome of surgery in patients with PC [15, 16]. The PCI may assess the tumor burden in the peritoneum, and it is used to guide patient selection for treatment. A high PCI score has been found to be a predictor of incomplete CRS and survival endpoints [16, 17, 18, 19, 20], and therefore may impact consideration of neoadjuvant therapy followed by reevaluation for CRS.

### 3.2 Cytoreductive Surgery

CRS is the removal of macroscopic peritoneal tumors performed by peritonectomy ± visceral resection and may require multiple procedures. It is considered the primary local therapy for patients with PC in the absence of extraperitoneal disease. Consideration of patterns of spread is important to plan the best surgical treatment. Three distinct patterns of PC spread have been identified [21]. Random proximal distribution (RPD) is a clinical scenario where peritoneal implantation is related to adherence of cancer cells, even in the presence of ascites. RPD is typically associated with intermediate to high‐grade tumors such as serous ovarian cancer, gastric cancer, CRC, and adenocarcinoma or carcinoid of the appendix, and may be treated with selective parietal peritonectomy of the macroscopically involved sites. Complete redistribution (CRD) is a pattern of spread that is not associated with adhesion to the peritoneal surface near the primary site. CRD is typically associated with less biologically aggressive tumor cells such as PMP and primary peritoneal mesothelioma and is treated with peritonectomy and an extended cytoreduction. The most aggressive type of peritoneal spread is widespread cancer distribution, which commonly presents with mucus‐producing cell surface adherence molecules that are typically associated with mucinous tumors of the ovary and colon/rectum, appendiceal cystadenocarcinomas, and various other undifferentiated mucin‐producing tumors, and requires a peritonectomy as an extended cytoreduction [21].

### 3.3 IP Therapy

As an adjunct to CRS, regional IP therapies are considered to achieve high drug exposure in the peritoneal cavity while sparing systemic toxicity with the goal of optimizing durability of local control and treatment outcomes [22]. A survival advantage associated with the use of IP therapy for PC has been demonstrated in multiple trials in selected patients [23, 24, 25, 26]. Notably, a limitation of IP therapy is the lack of efficacy in larger, bulky tumors. Postsurgical residual tumor size has been identified as the most significant prognostic indicator for efficacious IP therapy, with longer survival experienced in patients with smaller tumors (≤ 0.5 cm) compared with larger tumors (≥ 2 cm) [27]. Inadequate drug delivery to solid tumors is a major cause of treatment failure [28], hence, IP therapy should ideally be used in optimally surgically debulked cases where maximal residual tumor size is no > 2.5 mm for Hyperthermic IP chemotherapy (HIPEC) or 4 mm for Pressurized IP Aerosol Chemotherapy (PIPAC) [29]. Completeness of cytoreduction score (CCR) without residual tumor (CCR0) and completeness of cytoreduction with residual disease ≤ 2.5 mm (CCR1) are therefore best suited for IP therapy.

HIPEC involves the instillation of IP chemotherapy heated to 41°C–43°C into the peritoneal cavity, maintenance for 30 min–2 h, followed by drainage. HIPEC is used to enhance drug uptake and efficacy in the peritoneal cavity where systemic therapies have difficulty penetrating the blood‐peritoneal barrier [30, 31, 32]. Common chemotherapy agents utilized in HIPEC include mitomycin C, oxaliplatin, cisplatin, and doxorubicin. HIPEC can be administered via an open surgery approach or laparoscopically.

Preoperative IP treatments have been used to downstage bulky disease to facilitate CRS [33, 34]. Early postoperative IP chemotherapy (EPIC) is administered immediately following surgery over multiple consecutive daily doses. IP chemotherapy is delivered into the peritoneal cavity via an IP catheter and maintained for 4–24 h and followed by drainage [35, 36]. The advantage of HIPEC and EPIC is the opportunity of attaining even drug distribution in the cavity prior to the formation of tissue adhesions due to surgery [37]. EPIC offers several advantages over standard HIPEC, primarily due to increased drug contact time with the peritoneal surface. Unlike HIPEC, where the instilled solution covers only 30%–40% of the peritoneal surface if allowed for a short period, EPIC allows for prolonged contact with the peritoneum, resulting in more uniform drug distribution and enhanced cytotoxic effects.

Newer IP treatment modalities include Bidirectional/Neoadjuvant IP and Systemic Chemotherapy (BIPSC/NIPS) and PIPAC. BIPSC/NIPS is an aggressive IP treatment approach developed in Japan for patients with gastric cancer and PC. BIPSC/NIPS incorporates neoadjuvant systemic and IP chemotherapy followed by CRS, HIPEC, and EPIC with the goal of reducing tumor burden with neoadjuvant systemic and IP therapy, then removing residual macroscopic lesions with CRS and HIPEC, and to destroy residual microscopic peritoneal deposits with EPIC [38]. BIPSC/NIPS has limited availability in the United States.

PIPAC utilizes a minimally invasive laparoscopic approach with a nebulizer which pressurizes the drugs into aerosol. The pressurized delivery results in smaller doses, higher concentrations, and deeper penetration of IP drugs. It is considered a palliative treatment for patients with large tumor burden or considerable persistent ascites prohibitive of CRS ± HIPEC. PIPAC results in fewer chemical bowel perforations than HIPEC [39], it is contraindicated in patients with biliary or small bowel obstructions and extra‐abdominal metastasis [39]. The studies on the use of PIPAC in PC from intestinal, appendiceal, gastric, and ovarian cancers have emphasized its safety, better tolerability, and control on ascites production and is typically used in addition to systemic therapy [40, 41, 42]. More recently, indications for PIPAC treatment have been proposed beyond a purely palliative approach; however, studies investigating PIPAC in the prophylactic or adjuvant setting have demonstrated unknown benefit and may be associated with higher rates of toxicity due to deeper penetration into normal tissue after complete cytoreduction.

The peritoneal immune microenvironment is composed of extracellular matrix, endothelial cells, fibroblasts, lymphocytes, adipocytes, abundant dendritic cells, macrophages, and natural killer cells, and a high ratio of CD8+:CD4+ T cells. The peritoneum is known to induce T‐lymphocyte recruitment and proliferation, which provides the rationale for IP immunotherapy [43]. Various IP immunotherapies, including immunotoxins [44], immune stimulators [45], and chimeric antigen receptor (CAR)‐T cells [46] have been investigated in preclinical and clinical studies, some associated with improvement in overall survival (OS) [43]. The role of IP immunotherapy remains an active area of investigation.

### 3.4 Toxicities Associated With CRS ± IP Therapy

Patients with adequate performance status, disease confined to the peritoneal cavity, and limited tumor burden may be considered for CRS ± IP therapy; however, it may result in significant complications such as fistula formation, obstruction, and anastomotic leaks [47, 48]. Complications related to IP administration procedures include abdominal pain, infection, bleeding, perforation, blocked catheter, and port access difficulties [49, 50] as well as drug‐related toxicities such as hematologic toxicity, chemical peritonitis, and ileus [51, 52, 53, 54, 55, 56] have been reported; hence, aggressive local therapy should be considered in carefully selected patients with good performance status, in whom optimal cytoreduction is feasible. Age [57], tumor burden [58, 59], hypoalbuminemia [60], and performance status [61] have been identified as prognostic factors for morbidity and mortality associated with CRS and IP therapy and should be evaluated when considering treatment. Lastly, patient‐related quality of life (QOL) has been compared for patients receiving IP versus IV chemotherapy [62]. During active treatment, patients who receive IP therapy experience more health‐related QOL symptoms, abdominal symptoms, and neurotoxicity compared with patients receiving conventional IV therapy. However, only neurotoxicity remains significantly greater for IP patients 12 months following treatment [62]. It is important to discuss the impact of treatment options on QOL with patients undergoing therapy for PC.

### 3.5 Neoadjuvant/Adjuvant Therapy

Multimodality treatment regimens incorporating the addition of neoadjuvant or adjuvant systemic chemotherapy, targeted, immunotherapeutic agents, and immune checkpoint inhibitors to CRS with or without IP therapy have been investigated. These treatments are generally selected based on standard of care for tumor type or origin. More recently, molecular profile plays a role in the selection of matched targeted therapies in the era of precision oncology [63] (see Topic 1).

## 4 Summary of Literature Review

### 4.1 TOPIC 1/Impact of Histology and Molecular Subtype on Selection of Therapies With PC

We begin our discussion of the various therapeutic strategies for PC by describing the impact of different tumor histologies and molecular subtypes on multimodality treatment selection and outcomes for patients with PC. In contrast to most other sites, treatment of metastatic disease includes chemotherapy delivered directly to the peritoneal surface, in addition to systemic therapy (chemotherapy, immunotherapy, targeted therapy). The enthusiasm for IP chemotherapy relates to the poor perfusion of systemic treatments to the peritoneal surface as a result of the peritoneal‐plasma barrier [64]. While systemic therapy is beneficial in preventing metastases to other sites, it has limitations in the management of PC. IP chemotherapy in combination with systemic therapy is thus considered for patients with disease metastatic to the peritoneum.

#### 4.1.1 Subtopic 1/Selection of Systemic Therapy

The choice of systemic chemotherapy is dependent on the primary tumor site. Ovarian and fallopian tube carcinomas are typically treated with a platinum agent in combination with a taxane, while tumors of a gastrointestinal origin (gastric, CRC, appendiceal) are treated with 5‐fluoropyrimidine (5‐FU)‐based chemotherapy. Patients with tumors that respond well to systemic therapy tend to benefit most from systemic chemotherapy in addition to IP chemotherapy PC (i.e., ovarian cancer), whereas patients with less chemosensitive tumors (ex: low grade mucinous appendiceal carcinoma) tend to have little to no benefit from the addition of systemic chemotherapy to IP chemotherapy [65].

Bevacizumab is an important component of systemic therapy for the treatment of ovarian and certain gastrointestinal cancers; however, its role in the treatment of PC is controversial. The use of neoadjuvant bevacizumab, for example, is associated with an increased risk of surgical complications in both ovarian and colorectal cancer patients undergoing CRS [66, 67].

Systemic therapy regimens are typically dictated by the tissue of origin, as opposed to molecular subtype, with a few notable exceptions. For BRCA mutated ovarian cancer, adjuvant niraparib has demonstrated promising results in patients with ovarian cancer after CRS in a prospective non‐randomized trial [68]. For estrogen receptor positive low‐grade serous ovarian tumors, adjuvant estrogen blockade may lead to improved outcomes. In patients with PC mismatch repair deficient (dMMR) gastrointestinal cancers, immune checkpoint therapy has resulted in excellent response rates [69].

#### 4.1.2 Subtopic 2/Selection of IP Chemotherapy

Early studies of IP chemotherapy used either mitomycin C or cisplatin‐based regimens based on their high antitumor efficacy when used systemically. The choice of IP chemotherapy in the modern era has become increasingly dependent on tumor type. Multiple prospective trials in ovarian cancer have demonstrated that the use of IP chemotherapy with a cisplatin‐based regimen improves OS over CRS alone in patients with resected ovarian cancer who have received systemic therapy [26, 70, 71]. An improvement in 3‐year OS with the addition of cisplatin and 5‐FU based HIPEC to systemic therapy was also demonstrated in patients with PC from gastric cancer origin [72]. Similarly, an OS benefit was observed in patients with PC from gastric cancer who received neoadjuvant systemic therapy and HIPEC versus those that underwent CRS alone [73]. 5‐FU‐based IP chemotherapy has demonstrated benefit in patients with PC of CRC origin [23]. The PRODIGE 7 study randomized patients with PC from CRC to CRS versus CRS plus HIPEC (oxaliplatin‐ based) following systemic therapy. There was no OS benefit observed with the addition of HIPEC, which was associated with a higher rate of post‐surgical complications (42%, HIPEC vs. 32%, CRS alone) [74]. Proponents of HIPEC have argued that the 30‐min dwell time for the oxaliplatin was too short in this study and/or mitomycin C would have performed better in this setting. Of note, a previous retrospective review showed no differences in survival outcomes between oxaliplatin and mitomycin C‐based IP chemotherapy [75]. The ongoing GECOP‐MMC will evaluate mitomycin C in this context and should provide further clarity (NCT05250648).

#### 4.1.3 Subtopic 3/Timing of Systemic and IP Chemotherapy

Timing and benefit of systemic chemotherapy in combination with IP chemotherapy in patients with PC is controversial given the paucity of large randomized controlled trials to guide management. Hence, treatment with neoadjuvant and/or adjuvant systemic therapy is variable. For example, the timing of systemic therapy for CRC with respect to CRS and IP therapy is a topic of debate with limited data suggesting that adjuvant therapy confers higher survival compared to neoadjuvant therapy. The Cairo 6 trial [76] randomized patients with resectable PM from CRC to perioperative systemic therapy or CRS‐HIPEC alone and found that the proportions of macroscopic complete CRS‐HIPEC did not differ significantly between the two groups. Objective radiologic and major pathologic response rates of CPM to neoadjuvant treatment were promising at 28% and 38%, respectively, justifying further investigation in a phase III trial.

##### 4.1.3.1 Neoadjuvant Systemic Therapy

Data evaluating the role of neoadjuvant therapy prior to surgery for potentially resectable PC are mixed. While neoadjuvant chemotherapy offers early systemic treatment and may improve selection of patients for surgery, it may also lead to a decline in patient performance status or a missed window for surgery.

Previous investigations in patients with PC from ovarian cancer showed that receipt of neoadjuvant chemotherapy is associated with a higher rate of complete cytoreduction at the time of CRS [77, 78]. However, the benefit in improving completeness of surgical resection with neoadjuvant treatment did not directly translate into a survival benefit, raising questions about its importance [77, 79, 80, 81]. Interestingly, subgroup and pooled analyses of prospective studies have suggested that neoadjuvant chemotherapy may be associated with a greater benefit in those patients with high‐risk features, such as low serum albumin, worse baseline performance status, and Ca125 > 2000 [82, 83].

Other tumor types that have demonstrated a survival benefit to neoadjuvant systemic therapy prior to CRS and IP chemotherapy include gastric cancer [72] and CRC [84]. In contrast, the addition of neoadjuvant chemotherapy to IP chemotherapy has generally not demonstrated improved outcomes in patients with appendiceal cancers, particularly with goblet cell or signet ring tumors [85].

##### 4.1.3.2 Adjuvant Systemic Therapy

The rationale for use of adjuvant systemic therapy following CRS and IP therapy is to target microscopic residual tumor cells and potential subclinical metastatic disease. In diseases such as gastric and colorectal cancer in which systemic relapse is an important consideration, there is evidence suggesting that adjuvant chemotherapy may improve outcomes [76, 86]. In contrast, no benefit has been demonstrated from adjuvant treatment for cancers for which relapses are almost exclusively peritoneal, such as low‐grade serous ovarian tumors or low‐grade appendiceal tumors [87, 88].

It is important to consider the timing of adjuvant chemotherapy following IP therapy as delays in initiation have been associated with inferior survival outcomes; however, this may be related to poor post‐operative performance status as opposed to the impact of rapid adjuvant therapy initiation on disease outcomes [89, 90].

In conclusion, tumor site of origin, tumor histology, and molecular profile are important considerations in the selection of multimodality treatment for patients with PC. Selection of systemic treatments for patients with PC of various tumor types is becoming increasingly individualized, based on specific molecular subtype.

### 4.2 TOPIC 2/Management of PC From Gynecologic Malignancies

PC is frequently seen in patients with ovarian cancer, but is rare in endometrial and cervical cancer. Data from a Dutch Registry study including 94,981 patients diagnosed with ovarian, endometrial, or cervical cancer identified 61%, 2%, and 1%, respectively, presenting with PC. Predictors for PC in ovarian cancer included age 50–74 years, presence of distant metastases, poorly differentiated tumors, and serous histology. Predictors in endometrial cancer included positive lymph nodes, distant metastases, high‐grade tumors, and clear cell or serous histology. In cervical cancer, adenocarcinoma is associated with a higher risk of PC than squamous cell carcinoma [91]. PC from gynecologic tumor origin can either present as a result of metastasis or primary PC, which includes a variety of histologic subtypes including serous, mucinous, clear cell, undifferentiated, and endometrioid and is treated according to histology. PMP of gynecologic origin is most often associated with mucin‐producing ovarian tumors and is associated with poor outcomes. Herein we describe the data in support of CRS and IP therapy for the treatment of PC from gynecologic malignancies of different tumor origins.

#### 4.2.1 Subtopic 1/Ovarian/Fallopian Tube Epithelial Carcinomas (Variant Case 1)

Aggressive treatment of PC from tubo‐ovarian epithelial carcinomas with the goal of achieving a complete macroscopic resection followed by platinum‐based systemic chemotherapy has been reported in multiple trials [24, 25, 26, 29, 92]. In patients in whom initial cytoreduction is not feasible, the use of neoadjuvant platinum‐based systemic chemotherapy allows a high percentage of complete cytoreduction with acceptable and similar results to patients with initial surgery without neoadjuvant chemotherapy [83]. For platinum‐resistant tumors, targeted therapy with poly (ADP‐ribose) polymerase (PARP) inhibitors has been combined with chemotherapy to target specific molecular pathways in cancer growth and progression to enhance treatment efficacy with promising results [68, 93]. Herein we describe the data in support of treatment of PC of tubo‐ovarian epithelial origin.

##### 4.2.1.1 Role of CRS

Interval CRS is typically performed following neoadjuvant systemic therapy. A successful CRS depends on patient selection, the locations of tumors, and surgeon expertise. Patient performance status, histology, disease distribution, and dimension of residual disease have been identified as predictors of progression‐free survival (PFS) and OS [94]. The goal of CRS is to achieve maximal cytoreduction, with the goal of no residual disease [94]. Omentectomy should be performed as a component of CRS as the omentum is one of the most common metastatic sites of epithelial ovarian cancer. Infracolic omentectomy is a common procedure used during CRS but has been hypothesized to be associated with a higher risk of microscopic residual disease. A randomized trial compared infracolic omentectomy with infragastric omentectomy. Multivariate analysis revealed that infragastric omentectomy was associated with improved detection of omental metastases and a higher PFS in patients with stage IIB or higher disease and was not associated with a higher rate of perioperative complications. Hence, compared with infracolic omentectomy, infragastric omentectomy may be a more appropriate procedure for stage IIB–IIIC epithelial ovarian cancer patients with normal‐appearing omentum [95].

##### 4.2.1.2 Role of IP Therapy

Incorporation of IP therapy into an adjuvant chemotherapy regimen is considered an acceptable treatment option in carefully selected patients in whom maximal cytoreduction is achievable. Although data is lacking to support the use of IP therapy at the time of primary debulking, recent high‐quality studies have assessed the use of HIPEC for tubo‐ovarian cancer, mostly in the setting of interval surgery [96, 97, 98]. Most notably, the randomized OVHIPEC study demonstrated a benefit associated with the addition of cisplatin‐based HIPEC in patients undergoing interval CRS following neoadjuvant carboplatin and taxol for stage III ovarian cancer. This study reported a prolonged PFS (14.2 vs. 10.7 months without HIPEC) and 10‐year OS (45.7 vs. 33.9 months without HIPEC) [96]. However, the study has been criticized for small size (245 patients) and exclusion of stage IV disease. Another randomized study also confirmed an OS benefit associated with the addition of cisplatin‐based HIPEC to neoadjuvant chemotherapy followed by CRS in the interval setting (52 vs. 45 months without HIPEC) [97]. HIPEC was not associated with an increase in postoperative morbidity in either trial [96, 97]. A randomized trial published by Lim et al. found that the addition of HIPEC to CRS did not improve PFS or OS in a primary or interval surgery setting for all patients. However, a subgroup analysis demonstrated a PFS and OS advantage to the addition of HIPEC to interval CRS in patients who received neoadjuvant chemotherapy [98].

Data evaluating the benefit of HIPEC following CRS in the recurrent setting also appears to be inconclusive. A trial including patients with stage IIIC or IV ovarian cancer with recurrent disease who were randomized to second‐or third‐line chemotherapy versus HIPEC following debulking surgery demonstrated an improvement in 3‐year OS (75% with HIPEC vs. 18% *p* < 0.01) with no differences observed between platinum‐resistant and platinum‐sensitive tumor recurrences [99]. In patients who did not receive HIPEC, CRS was associated with longer survival. Patients with a PCI score of < 15 also appeared to have longer survival [99]. Conversely, a more recent single institution study included 98 patients with platinum‐sensitive recurrent PC randomized to carboplatin‐based HIPEC after CRS followed by 5–6 cycles of carboplatin‐based systemic therapy. HIPEC with carboplatin was well tolerated but did not result in superior survival outcomes (median PFS 12.3 months with HIPEC vs. 15.7 months with HIPEC + carboplatin, *p* = 0.05; median OS 52.5 months with HIPEC vs. 59.7 months with HIPEC and carboplatin, *p* = 0.31) [100].

Studies have also evaluated the potential benefit of EPIC in patients with advanced ovarian cancer. Although there is less data available, one randomized study including 218 patients with FIGO IIIC‐IV disease who were randomized to dose‐dense EPIC followed by IV chemotherapy or IV chemotherapy alone demonstrated that dose‐dense EPIC was significantly associated with a prolonged 5‐year PFS (26%, vs. 8.5%, respectively) and median OS (67.5 vs. 46.3 months) compared with IV chemotherapy alone [101].

In summary, despite some encouraging published results, trials have failed to consistently show a benefit to IP therapy in patients with PC from tubo‐ovarian epithelial cancers. Careful attention to patient selection and ability to achieve maximal CRS should be used when considering this approach. There continues to be debate about the advantages of primary versus interval surgery with IP therapy. The TRUST trial (NCT02828618) is designed to assess the potential superiority of primary vs. interval CRS. Future studies will further investigate whether certain histologies or molecular profiles gain particular benefit from its use, particularly for homologous recombination deficiency (HRD) and BRCA wild‐type tumors that appeared to have the greatest benefit in the OVHIPEC study [102].

##### 4.2.1.3 Patterns of Recurrence Following CRS ± IP Therapy

There is a high risk of systemic failure in women with advanced or recurrent tubo‐ovarian epithelial cancer treated with CRS and IP therapy, with one‐half experiencing their first recurrence outside of the peritoneal cavity [103]. For interval CRS and IP therapy, recurrence locations were pelvic (50%), upper abdomen (23%), and extraperitoneal (57%). While for recurrent disease treated with CRS and IP therapy, patients experienced recurrences in the pelvis (23%), upper abdomen (5%), and extraperitoneal location (60% of which 67% presenting with extraperitoneal recurrence alone) [103].

#### 4.2.2 Subtopic 2/Pseudomyxoma Peritonei (PMP) (Ovarian Origin)

PMP of ovarian origin is a relatively rare, progressive, slowly growing cancer that is associated with poor outcomes. Ovarian PMP can be classified as mucinous ovarian neoplasms (20%), mucinous neoplasm in ovarian teratoma (26.7%), mucinous lesion probably arising in ovarian teratoma (33%), and non‐specific (20%) [104]. The most frequent somatic gene mutations associated with PMP include KRAS (38%–100%), GNAS (17%–100%), and TP53 (5%–23%); however, there are conflicting results correlating these with survival [105]. Herein, we describe the data in support of treatment of PMP of ovarian origin.

##### 4.2.2.1 Role of CRS

Optimal cytoreduction is important when considering CRS for PMP. Total parietal peritonectomy can be used to treat peritoneal surface cancers and subsequent recurrences. Completeness of cytoreduction score (CCR) without residual tumor (CCR0) and completeness of cytoreduction with residual disease ≤ 2.5 mm (CCR1) and intraoperative PCI score < 21 have demonstrated prognostic significance for OS following CRS in PMP [106]. CRS should be considered in patients with adequate performance status to tolerate surgery and who are likely to achieve optimal cytoreduction (CCR0–1).

##### 4.2.2.2 Role of IP Therapy

The combination CRS and HIPEC is considered a standard of care for the treatment of PMP despite the lack of randomized data. In general, responses to standard‐of‐care chemotherapy at the time of recurrence or progression of mucinous ovarian cancer are relatively poor. Several potential explanations for the limited benefit of HIPEC in this setting include low cell proliferative activity and mucin overproduction [104]. However, given the rarity of mucinous ovarian cancer and the lack of effective systemic treatment options, HIPEC may be worth considering. It remains unclear whether HIPEC is best given at the time of initial diagnosis, as a second staging surgery following the initial diagnosis in the up‐front setting, or at the time of secondary debulking. Additionally, iterative CRS and HIPEC can be used in selected cases of recurrence. The retrospective French RENAPE Group study results suggested that CRS plus HIPEC used as a primary therapeutic strategy holds promise in patients with ovarian PMP showing a difference in DFS (*p* = 0.0463) between those with teratoma/likely‐teratoma origin (100% at 1, 5, and 10 years) and mucinous ovarian epithelial neoplasms or nonspecific subtypes (100%, 66.6%, and 50% at 1, 5, and 10 years, respectively) [104].

##### 4.2.2.3 Patterns of Recurrence Following CRS ± IP Therapy

In patients with PMP from ovarian origin treated with CRS and IP therapy, the primary site of recurrence is peritoneal, especially in patients with mucinous tumors [107].

#### 4.2.3 Subtopic 3/PPC of Gynecologic Origin (Variant Case 2)

PPC is a rare cancer of the peritoneum associated with poor prognosis. It is estimated that up to 15% of women diagnosed with advanced ovarian cancer have PPC. PPC is classified based on histology as EOPPC, serous surface papillary carcinoma, papillary serous carcinoma of the peritoneum, extraovarian Mullerian adenocarcinoma, and normal‐sized ovarian carcinoma syndrome. EOPCC is associated with BRCA‐1 or BRCA‐2 germline mutations in 18% of cases. These are serous carcinomas in 90% of cases and occur in women with a mean age at diagnosis of 56–62 years [108]. Herein we describe the data in support of the treatment of PPC of gynecologic origin.

##### 4.2.3.1 Role of CRS

PPC is managed similarly to serous ovarian carcinomas, with CRS combined with systemic chemotherapy and targeted therapy [8]. The goal of surgery is to provide optimal cytoreduction typically achieved with hysterectomy with bilateral salpingo‐oophorectomy and omentectomy, which are performed in all cases to remove the primary tumor and any involved tissues. Platinum‐based systemic therapy is typically recommended in the neoadjuvant or adjuvant setting [8, 109]. For platinum‐resistant tumors, PARP inhibitors combined with chemotherapy are considered [68, 93].

##### 4.2.3.2 Role of IP Therapy

Retrospective data [110] and a phase III trial have demonstrated that IP chemotherapy is associated with improved survival compared to IV chemotherapy alone in patients with PPC of gynecologic origin [26]. Best results are achieved for CCR0–1 resections [8]. Iterative CRS and HIPEC can also provide benefit in selected cases of recurrence.

##### 4.2.3.3 Patterns of Recurrence Following CRS ± IP Therapy

Like PMP, the peritoneum is the primary site of recurrence for patients with PPC treated with CRS and IP therapy, with incidences varying with tumor subtype and extent of cytroreduction [103].

#### 4.2.4 Subtopic 4/Other Gynecologic Malignancies (Uterine Cancer and Uterine Carcinosarcoma)

PC from primary or recurrent endometrial carcinoma is common and is associated with poor survival, and the optimal treatment remains unclear.

##### 4.2.4.1 Role of CRS

Retrospective studies reported outcomes for patients undergoing CRS for recurrent endometrial carcinoma. In each of these studies, longer median OS [111, 112, 113] and 5‐year OS [114, 115] were observed in patients with optimal cytoreduction compared to patients with residual disease. The survival advantage was also demonstrated in a meta‐analysis of 672 patients from 14 retrospective cohorts who underwent complete cytoreduction [116]. A prospective phase II study evaluating the feasibility of neoadjuvant chemotherapy, followed by debulking surgery, for clinically diagnosed FIGO stage IVb endometrial cancer demonstrated a median DFS of 9.1 months and median OS of 23.2 months [117]. CRS for recurrent endometrial cancer may provide an opportunity for long‐term survival in select patients.

##### 4.2.4.2 Role of IP Therapy

The use of CRS and HIPEC for managing PC from primary and recurrent endometrial carcinoma is limited. Retrospective series suggest a survival advantage when HIPEC is delivered following CRS when optimal cytoreduction is achieved [118, 119, 120]. A systematic review identified 14 retrospective series reporting on the role of HIPEC in recurrent endometrial cancer and 12 retrospective studies for uterine carcinosarcoma. Altogether, HIPEC seems to play a significant role in the treatment of mostly recurrent uterine leiomyosarcoma with peritoneal sarcomatosis and found CRS plus HIPEC is associated with improved survival and lower peritoneal recurrence rates [121]. In these studies, completeness of CRS, PCI score, histologic subtype, or selection of systemic therapy all influence HIPEC effectiveness [121]. Further investigation is warranted for the use of HIPEC in the setting of recurrent uterine cancer and uterine carcinosarcoma.

### 4.3 TOPIC 3/Management of PC From Gastrointestinal Malignancies

The peritoneal surface is a common site of spread for patients with gastrointestinal tumors. Patients with CRC present with PC in approximately 10% of all cases [122] and 40% of patients with recurrent CRC present with PC without evidence of other distant metastases [123]. Appendiceal carcinomas are relatively rare tumors. A common pathway of dissemination of all appendiceal tumors, regardless of grade and cell of origin, involves invasion of the appendiceal wall, luminal obstruction, and perforation with subsequent dissemination throughout the peritoneal cavity [124]. Patients with appendiceal mucinous neoplasms, similar to patients with neoplastic mucinous tumors of gastric, ovarian, pancreatic, and colorectal origin, are at risk for pseudomyxoma peritonei (PMP) syndrome [125]. Peritoneal metastasis is present in 5%–30% of the patients undergoing potentially curative surgery for gastric cancer [126, 127], and is found in 30%–40% at the time of diagnosis [128]. Herein, we describe the role of CRS and IP therapy for treatment of PC from gastrointestinal malignancies of different tumor origins.

#### 4.3.1 Subtopic 1/Colorectal Cancer (CRC) (Variant Case 3)

The prognosis of patients with PC from CRS is inferior to that of patients with PC from other metastatic sites [129], with average survival < 9 months for untreated patients and up to 24 months for those that receive systemic therapy [130], providing a rationale for assessing the role of IP therapy.

##### 4.3.1.1 Role of CRS

In select patients with PC from CRC, the addition of CRS to partial colectomy to remove the primary tumor can be considered. Primary CRS (with or without IP therapy) or interval CRS following neoadjuvant systemic therapy can be considered. Optimal candidates for consideration of CRS include extent of peritoneal involvement and patient performance status. Multiple organ resections and partial to subtotal parietal peritonectomy are often required for optimal cytoreduction, and omentectomy is often necessary to better explore the abdominal and pelvic peritoneal cavity. Studies report a relative contraindication for CRS associated with PCI score > 15 [131] or 17 [132] for CRC PC patients.

The effectiveness of CRS is evaluated using the CCR score quantifying the extent of residual disease [133]. Complete macroscopic cytoreduction (CCR0–1) is the basis for successful multimodal therapy.

##### 4.3.1.2 Role of IP Therapy

Numerous studies have reported the efficacy of HIPEC following CRS for treatment of PC in patients with CRC [134, 135, 136, 137, 138, 139, 140]. The addition of CRS and IP therapy has demonstrated improvements in survival [23] and, in properly selected patients, median OS of up to 51 months [137, 141]. Long‐term remissions have been reported in select patients with low PCI (≤ 10) who undergo complete CRS and IP therapy [142]. Notably, a large Dutch study of over 37,000 study patients treated with CRS and HIPEC in addition to systemic therapy demonstrated a doubling of survival compared to systemic therapy alone (12.5 vs. 6 months, *p* < 0.0001) [143]. However, the data from this report was obtained over a 20‐year period (1995–2000‐systemic treatment alone; 2010–2014‐addition of CRS and HIPEC) thus, reasons for this improvement may also be due to improvements in systemic therapy. Four recent important randomized trials have addressed the role of HIPEC in the treatment of CRC PC [74, 144, 145, 146].

The UNICANCER PRODIGE 7 randomized trial examined the benefit of adding oxaliplatin‐based HIPEC to CRS compared with CRS alone in 265 patients with PCI of ≤ 25 [74]. The HIPEC regimen used in this trial included bidirectional therapy with IV 5‐FU and Folinic acid 20 min prior to IP. All patients were required to complete at least six cycles of systemic chemotherapy before or after surgery. The study did not detect a difference in median OS (41.7 months for CRS plus HIPEC group and 41.2 months for CRS alone *p* = 0.99). There was higher grade‐3 morbidity observed within 60 days in the HIPEC arm (26% vs. 15% *p* = 0.035). This study concluded that the addition of HIPEC to CRS does not result in a survival benefit compared to CRS alone in patients with CRC PC [74]. However, the study would support the working hypothesis that CRS with the goal of no residual disease results in the best outcomes, and a specific subgroup of patients (i.e., those with a PCI of 11–15) might benefit most.

Similarly, the PROPHYLOCHIP randomized trial investigated the potential role of second‐look surgery with HIPEC in patients with CRC considered at high risk of peritoneal recurrence who had synchronous PC or ovarian metastases initially resected with the primary tumor, or perforated tumors [144]. Patients received 6 months of adjuvant chemotherapy, and if there was no evidence of disease recurrence on imaging, they were randomized to surveillance vs. second‐look surgery (with CRS and HIPEC if intraoperative evidence of PC recurrence). HIPEC regimens used in this study included IP oxaliplatin, IP oxaliplatin plus irinotecan and intravenous (IV) 5‐FU, IP mitomycin‐C alone (in cases of neuropathy), or IP oxaliplatin and IV 5‐FU. No statistically significant differences in 3‐year disease‐free survival (DFS) (44% CRS+ HIPEC vs. 53% surveillance, *p* = 0.82) or 5‐year OS (68% and 72%, respectively) were detected, but significant rates of postoperative morbidity were associated with second‐look surgery and HIPEC [144]. The study highlights that a proactive strategy, including a systematic second‐look surgery plus HIPEC, failed to improve survival in comparison to adequate surveillance.

The HIPECT4 multicenter randomized trial [146] evaluated the role of HIPEC using mitomycin‐C in the prevention of peritoneal recurrence in patients with T4 CRC and demonstrated an improved 3‐year local control rate compared with CRS alone (97.6% and 87.6%, respectively; *p* = 0.03), no differences in DFS or OS were observed.

Lastly, the randomized COLOPEC trial evaluated adjuvant and prophylactic HIPEC in patients with advanced colon cancer (T4NxM0) or perforated disease without evidence of PC [145]. Patients were randomized to adjuvant oxaliplatin‐based HIPEC followed by systemic chemotherapy (5‐FU and leucovorin) or adjuvant systemic chemotherapy alone. No difference in 18‐month peritoneal metastasis‐free survival was detected between groups (80.9% for the HIPEC group vs. 76.2% for the systemic therapy alone group *p* = 0.28) as determined by diagnostic laparoscopy performed at 18 months. Peritoneal metastases were treated with CRS plus HIPEC in 68% of patients in the HIPEC group and 65% of the systemic therapy only group [145]. Long‐term results continued to demonstrate no differences in outcome with 5‐year peritoneal metastases rates at 63.9% and 63.2% (*p* = 0 0.907) and 5‐year DFS rates at 55.7% and 52.3% (*p* = 0.875), respectively [147]. Similarly, one recently reported randomized trial demonstrated that the addition of mitomycin C‐based HIPEC to complete surgical resection and adjuvant chemotherapy for cT4NXM0 colon cancer improved the 3‐year local control (LC) rate compared with surgery alone (97.6% vs. 87.6% *p* = 0.03) however, this benefit did not translate into a survival benefit (91.7% vs. 92.9%, *p* = 0.68) [146].

Criticisms of these studies have focused on the fact that all three of these trials tested single‐agent oxaliplatin over a duration of 30 min in a selected group of patients. The results of these trials raised the question of whether IP oxaliplatin is the best choice for HIPEC for patients with CRC, and considering that only one‐half of oxaliplatin is systemically absorbed over 30 min, perhaps extending exposure to IP oxaliplatin over time might improve HIPEC efficacy. Future trials focus on the addition of a second drug to IP oxaliplatin (NCT02974556), or shifting to mitomycin C‐based HIPEC (HIPECT4, NCT02614534; GECOP‐MMC, NCT05250648).

The role of EPIC has been investigated in patients with CRC PC with mixed results. A randomized trial examined the benefit of EPIC in patients with CRC PC. Patients underwent complete CRS followed by EPIC and systemic chemotherapy or chemotherapy alone. In this study, the use of EPIC did not result in a survival benefit, and complete resection of PC resulted in a 2‐year OS rate of 60% [148]. Another retrospective study analyzed the outcomes of CRC PC patients who underwent CRS without IP therapy compared with those of patients who received CRS with EPIC or HIPEC. In this study, the use of both EPIC and HIPEC was statistically significantly associated with better cause‐specific survival (CSS) and PFS compared to CRS alone. CSS was also significantly associated with perioperative systemic chemotherapy and was independently related to the completeness of cytoreduction score (CCR) in patients with both peritoneal and extraperitoneal metastases [149].

In summary, the role of CRS and IP therapy for patients with CRC and PC remains a topic of investigation and may be considered in select patients.

##### 4.3.1.3 Patterns of Recurrence Following CRS ± IP Therapy

In patients with PC from CRC undergoing CRS and IP therapy, failure rates are high. Isolated peritoneal recurrences occur in approximately 25%, isolated hematogenous recurrence is found in 28%, and mixed recurrence occurs in 14% of patients [150]. On multiple logistic regression analysis, BRAF and KRAS mutational status and positive lymph nodes have been identified as predictive factors for peritoneal recurrence [150].

#### 4.3.2 Subtopic 2/Appendiceal Carcinomas (Variant Case 4)

Outcomes for patients with PC from cancer of appendiceal origin depend primarily on histology, extent of peritoneal involvement, completeness of resection, and patient performance status [151, 152]. Appendiceal tumors are typically grouped based on mucin production, and in general, mucin‐producing appendiceal neoplasms are associated with a less aggressive biologic behavior than nonmucinous tumors [153, 154]. Patients with low‐grade mucinous tumors typically progress slowly and may develop PMP associated with mucinous ascites. Approximately 7% are associated with lymph node involvement, and up to 16% will dedifferentiate into higher‐grade tumors [155]. High‐grade appendiceal mucinous tumors are more likely to behave aggressively with metastases and are associated with worse prognosis [156].

PMP from appendiceal origins are classified into the following types: disseminated peritoneal adenomucinosis (DPAM), peritoneal mucinous carcinomatosis (PMCA), and PMCA with intermediate or discordant features [157]. Clinical outcomes vary depending on tumor type; however, the most important prognostic factor remains the extent of cytoreduction [157, 158]. DPAM has been further classified as well‐differentiated mucinous carcinomatosis and low‐grade mucinous appendiceal neoplasms (low‐grade mucinous carcinoma peritonei), while high‐grade mucinous carcinoma peritonei includes moderately or poorly differentiated adenocarcinomas, PMCA, and cases with signet‐ring cell component. High‐grade mucinous carcinoma peritonei are associated with worse survival, especially in patients with high PCI indices [152, 159, 160], whereas patients with low‐grade mucinous tumors are associated with a better prognosis but do not appear to derive as much benefit from the addition of systemic therapy to IP treatment [161].

##### 4.3.2.1 Role of CRS

In addition to appendectomy, a thorough examination of the abdomen is required to assess for evidence of peritoneal metastases prior to proceeding with CRS in patients with suspected or proven peritoneal involvement. In cases where a perforated mucinous adenocarcinoma and/or peritoneal disease is discovered after appendectomy, a right‐sided hemicolectomy with CRS is recommended for completeness of staging. The completion of CRS is the most important prognostic factor for survival in patients with appendiceal carcinoma PC [151]. Complete CRS is defined differently for low‐grade mucinous and high‐grade appendiceal neoplasms. CCR0–1 resections are associated with similar outcomes for low‐grade mucinous tumors, while for high‐grade appendiceal tumors, CCR1 resections are associated with worse survival than CCR0 resections, and similar survival with CCR2 resections [152, 155]. The PCI at the time of CRS also appears to be prognostic for OS even in cases where a complete cytoreduction is achieved. Elias et al. [152] reported a 5‐year survival rate of 57% for patients with PMP with CCR0 and PCI score > 19, compared to 83% for patients with CCR0 and PCI < 19 (*p* = 0.004). PCI is also predictive of the ability to achieve CCR0 in high‐grade appendiceal tumors; however, there is no known PCI threshold associated with a survival advantage [162]. CRS should be considered for high‐grade appendiceal tumors as long as complete CRS (CCR0) is feasible and safe. In patients with high‐grade appendiceal cancers in whom CCR0 is deemed unlikely, CRS should not be attempted, as it offers no survival benefit and may be associated with operative morbidity and delays in receiving systemic therapy, if indicated [155].

Approximately 10% of all appendiceal patients who undergo CRS with or without HIPEC ultimately require repeat CRS for recurrent peritoneal disease. The completeness of second CRS [155, 163] and the interval between procedures [107, 164] is prognostic for survival. Incomplete initial CRS is not an absolute contraindication for second CRS for low‐grade appendiceal primaries, as 20% of these patients achieve a complete CCR0 or CCR1 during the second CRS [165], it is unlikely to achieve CCR0 following repeat CRS for high‐grade appendiceal tumors [164].

##### 4.3.2.2 Role of IP Therapy

The combination of CRS and IP therapy (HIPEC or EPIC) is considered the best treatment for patients with PC from appendiceal origin, even in the setting of incomplete cytoreduction where patients can still experience improved survival and symptom control. Several retrospective studies have evaluated the benefit of adding HIPEC to CRS in patients with PC of appendiceal origin [152, 153, 166, 167]. This benefit appears to vary according to PCI [159], extent of cytoreduction [159, 167], presence of nodal involvement [155, 167], and tumor grade [152, 153, 167]. One retrospective study reported that negative predictors of survival following CRS and HIPEC in patients with low‐grade appendiceal PC included positive nodal status (*p* = 0.003), incomplete cytoreduction (*p* < 0.0001), and preoperative chemotherapy (*p* = 0.04). For patients with high‐grade tumors, incomplete cytoreduction (*p* = 0.0003) and preoperative chemotherapy (*p* = 0.0064) were negative predictors of survival [155]. Despite this variability in benefit associated with HIPEC, it should be considered in patients with appendiceal cancer who achieve CCR0 or CCR1 during CRS.

There are data to support the use of HIPEC and EPIC following CRS in patients with PC from appendiceal carcinoma with reports of improved PFS and OS [168, 169, 170, 171, 172, 173]. An international registry including over 2000 appendiceal cancer patients demonstrated a median survival of 16.3 years and a 10‐year survival of 63% associated with CRS and HIPEC used in this setting [168]. An international cohort study compared the survival of patients with PMP of appendiceal origin treated with CRS with or without HIPEC. In this study of 1924 patients, the addition of HIPEC after CRS was associated with a significantly better 5‐year OS (58% HIPEC vs. 46% CRS alone) and did not result in increased post‐operative complication [174]. Hence, CRS and HIPEC are considered a standard of care in patients with low‐grade appendiceal neoplasms associated with PMP in patients who are deemed able to tolerate the procedure [168, 169, 170, 171].

EPIC has also been used for the treatment of low‐grade appendiceal mucinous neoplasms including PMP; however, there are no phase III data comparing EPIC with HIPEC, and literature is limited. In a propensity score‐matched analysis including 52 patients with low‐grade appendiceal mucinous neoplasms, it was demonstrated that patients who were treated with HIPEC + EPIC had an improvement in median OS of 34.3 months (127.3 vs. 93.0 months, *p* = 0.02). Median length of stay was higher in those who received EPIC compared to HIPEC alone (25.0 vs. 23.5 days, *p* = 0.028) [175]. Other studies show that EPIC and HIPEC have similar perioperative and long‐term outcomes in patients with mucinous appendiceal carcinoma presenting with PC who undergo CRS [176, 177]. Limited data supports the use of EPIC for the treatment of low‐grade appendiceal mucinous neoplasms including PMP; however, further studies are warranted examining the benefits. The ongoing randomized ICARUS (NCT01815359) is designed to further assess the role of EPIC and HIPEC in patients with appendiceal and CRC with isolated peritoneal metastases following optimal CRS.

##### 4.3.2.3 Patterns of Recurrence Following CRS ± IP Therapy

In all patients with PC from appendiceal carcinoma treated with CRS and HIPEC, progressive peritoneal disease is the most common site of recurrence, especially in patients with mucinous tumors. The median survival after peritoneal‐only recurrence is reported to be 33 months (95% CI 27.8–38.9 months), and patients who undergo repeat CRS survive longer than those who received systemic chemotherapy alone. High‐grade tumors can also give rise to hematogenous metastases [178].

#### 4.3.3 Subtopic 3/Gastric Cancer (Variant Case 5)

The majority of gastric cancer patients are diagnosed with advanced disease. Peritoneal spread is found at the time of diagnosis in 30%–40% of cases [128] and peritoneal relapses occur in approximately 40% of patients who are originally treated with potentially curative surgery and systemic therapy [128]. In patients with PC from gastric cancer, the presence of ascites is associated with a poor prognosis. Median survival with palliative chemotherapy for metastatic gastric cancer with PC is 6 months [128] however, select patients with adequate performance status and who are candidates for potential complete cytoreduction may benefit from CRS and HIPEC following neoadjuvant therapy. Eligibility can be determined with laparoscopic staging with peritoneal lavage in advanced gastric cancer.

##### 4.3.3.1 Role of CRS

For patients with PC from gastric cancer, complete CRS removing all macroscopic tumor deposits is essential to achieve good outcomes. Despite surgical removal of all macroscopic tumor, microscopic residual disease is not addressed with CRS alone, and perioperative systemic chemotherapy does not penetrate the blood‐peritoneal barrier well [128]. This provides a rationale for direct delivery of IP therapy to enhance local control following CRS.

##### 4.3.3.2 Role of IP Therapy

###### 4.3.3.2.1 Prophylactic IP Therapy Following Potentially Curative Gastrectomy

In patients with locally‐advanced gastric cancer without PC, adjuvant HIPEC following potentially curative gastrectomy has been shown to have potential in preventing peritoneal relapse, especially in certain aggressive histological subtypes of gastric cancer such as signet‐ring cell tumors, which occur in 30%–40% of cases and frequently develop peritoneal disease [128]. Several studies have demonstrated the effectiveness of HIPEC as a prophylactic treatment, resulting in improved survival rates compared to patients who are treated with gastrectomy without prophylactic HIPEC [179, 180, 181, 182]. Studies that specifically address the effectiveness of HIPEC in signet‐ring gastric cancers include a randomized trial that included 12% of patients with signet‐ring tumors in the HIPEC group [183] and a prospective non‐randomized study of CRS and HIPEC including patients with signet‐ring tumors [184] both showing that the presence of signet‐ring cells was not a poor prognostic factor and should be considered for treatment with CRS and HIPEC with limited peritoneal disease.

In a study by Yonemura et al., patients with localized gastric cancer were randomized to one of three treatment groups (surgery alone, surgery followed by IP chemotherapy (normothermic), or surgery followed by HIPEC). The HIPEC group was associated with a 5‐year OS rate of 61%, vs. 44% with normothermic IP chemotherapy, and 42% with surgery alone (*p* = 0.021). Major operative complications and mortality rates were similar between groups [179]. Similarly, another series compared 51 patients with cT4N0‐3M0 gastric cancer who were treated with HIPEC and systemic chemotherapy with 62 patients who were treated with systemic chemotherapy alone. The 3‐year OS rate was significantly higher in the HIPEC group (69% HIPEC vs. 66% systemic chemotherapy; *p* = 0.044), with only 3.9% of patients in the HIPEC group developing peritoneal recurrence, compared to 17.7% in the systemic therapy alone group. No significant difference in postoperative or chemotherapy complications was observed between groups [180]. Other studies have demonstrated similar benefits associated with prophylactic HIPEC in addition to potentially curative gastrectomy and systemic therapy [181, 182].

An early randomized trial examined the benefit of EPIC for the treatment of resectable gastric cancer without PC in which 248 patients were treated with D2 gastrectomy with or without 5‐FU and mitomycin C‐based EPIC. An improvement in 5‐year OS with gastrectomy + EPIC (54%) was observed compared to gastrectomy alone (38%, *p* < 0.05). The authors of this study identified two subsets of patients that had a statistically significantly increased OS associated with the use of EPIC, those with gross serosal invasion and lymph node metastasis, and proposed these as selection criteria for the use of EPIC in advanced gastric cancer [185]. Another retrospective study in patients with resectable gastric cancer demonstrated a similar survival advantage associated with EPIC and reduced the risk of peritoneal recurrence for the EPIC group (18.5% and 32.2%, respectively, *p* < 0.05) [186]. In contrast, the INPACT randomized phase II trial did not demonstrate a benefit when comparing intraoperative IP paclitaxel and post‐operative IV paclitaxel for the treatment of resectable nonmetastatic gastric cancer with high risk of PM following gastrectomy, though the IP therapy was not the traditional EPIC regimen [187].

In summary, prophylactic HIPEC following gastrectomy can be considered in locally advanced gastric cancer patients at high risk for peritoneal recurrence to reduce the risk of PC and prolong survival; however, this practice has not been universally adopted as standard of care. Ongoing trials evaluating prophylactic HIPEC include the phase III GASTRICHIP (NCT01882933) and GOETH (NCT03917173) studies that compare gastrectomy with and without HIPEC for advanced gastric cancer patients. Similarly, prophylactic EPIC can be considered in select patients (gross serosal invasion and lymph node metastases); however, there are no phase III data comparing EPIC with HIPEC, and further studies examining the prophylactic use of EPIC for the treatment of gastric cancer PC are warranted.

###### 4.3.3.2.2 IP Therapy for Gastric Cancer Patients With PC

For patients with PC from gastric cancer who have adequate performance status and are deemed operative candidates, neoadjuvant chemotherapy and CRS followed by HIPEC may be considered. Retrospective and case–control studies demonstrate encouraging results for patients with gastric cancer and PC treated with CRS and HIPEC [184, 188, 189, 190]. The CYTO‐CHIP propensity score‐matched retrospective study compared the outcomes of 180 patients with PC from gastric cancer treated with CRS (no residual tumor deposits > 2.5 mm) and HIPEC with 97 similar patients treated with CRS alone. Median survival for the HIPEC group was 18.8 vs. 12.1 months for the CRS alone group [73]. In addition, a randomized trial that included gastric cancer patients with PC treated with CRS with or without HIPEC (mitomycin C with cisplatin) resulted in a significantly longer median survival (11 vs. 6.5 months, respectively) and 3‐year OS rate (5.9% vs. 0%, respectively) associated with the use of HIPEC [183].

Despite these results, CRS and HIPEC are not universally considered a standard of care treatment regimen for gastric cancer patients with PC, owing largely to the absence of randomized clinical trials. Several ongoing phase III trials are evaluating the use of HIPEC in addition to CRS for gastric cancer patients with PC. The recently published GASTRIPEC (NCT02158988) showed no difference in median survival with or without the addition of HIPEC to CRS; however, in the subset of patients that obtained complete CRS, a significant increase in 5‐year OS associated with the use of HIPEC was observed (10% vs. 0%) [191]. In the Dutch phase III PERISCOPE II study (NCT03348150), gastric cancer patients with limited PC and/or positive peritoneal cytology are randomized to gastrectomy, CRS, and HIPEC versus current standard treatment (palliative systemic chemotherapy). The PERISCOPE trial has enrolled 182 patients, with estimated completion in 2026 [127]. Future publication of these studies may provide additional insights into the value of CRS and HIPEC in this setting.

The use of EPIC in the treatment of gastric cancer patients with PC has also been reported with inconclusive results. A prospective nonrandomized study reported a median OS of 11.4 months for patients who underwent D2 gastrectomy and CRS followed by 5‐FU and mitomycin C‐based EPIC. There was a significant survival difference demonstrated between patients who underwent CCR0 CRS (25.5 months), CCR1 (15.6 months), and CCR2 (7.2 months) (*p* < 0.05) [192]. A French retrospective multicentric study reported outcomes for 150 patients who received EPIC for PC from gastric cancer. Median OS was 9.2 months and 1‐, 3‐, and 5‐year survival rates were 43%, 18%, and 13%, respectively. The only independent prognostic factor identified by multivariate analysis was the completeness of CRS. In cases when complete CRS was achieved, the median survival was 15 months with a 1‐, 3‐, and 5‐year survival rate of 61%, 30%, and 23%, respectively [190]. Further studies examining the use of EPIC for the treatment of gastric cancer PC are warranted.

##### 4.3.3.3 Patterns of Recurrence Following CRS ± IP Therapy

Locoregional recurrence and distant metastases are common in gastric cancer treated with curative intent, with higher rates of distant metastases observed in patients with poor response to systemic therapy [193]. In patients with gastric cancer and PC treated with CRS, the most common site of recurrence is peritoneal, with the addition of HIPEC significantly reducing peritoneal recurrence rates, particularly in patients with lower PCI scores correlating with a lower tumor burden [183].

### 4.4 TOPIC 4/Management of PC From Other Histologies

Herein we describe the data in support of treatment of PC from rare histologies.

#### 4.4.1 Subtopic 1/Primary Peritoneal Mesothelioma (Variant Case 6)

Primary peritoneal mesothelioma is a rare tumor of peritoneal origin which accounts for approximately 30% of all mesotheliomas [194]. Early diagnosis is often difficult because the early symptoms are often overlooked, and most cases are diagnosed at an advanced stage when the disease is widespread throughout the peritoneal cavity. Primary peritoneal mesothelioma commonly presents with diffuse, extensive spread throughout the abdomen with rare metastatic spread beyond the abdominal cavity [194]. Treatment approaches have evolved from palliative systemic therapy or surgery to aggressive CRS and perioperative IP chemotherapy [194]. The best treatment option for a medically operable patient in whom cytoreduction is needed includes CRS and IP therapy (HIPEC or EPIC). Even with incomplete cytoreduction, patients can experience improved survival and symptomatic control. Early diagnosis and aggressive treatment with surgery and HIPEC can result in 5‐year survival rates of up to 65%, and without treatment, the average life expectancy is approximately 6 months.

##### 4.4.1.1 Role of CRS

Due to the low incidence of primary peritoneal mesothelioma, there have been no randomized studies to define optimal treatment. Since diffuse abdominal peritoneal involvement characterizes this disease, aggressive local therapy with optimal cytoreduction with or without IP therapy is a reasonable approach in patients who can tolerate the procedure. Consistent with data from other PC histologies, tumor burden, PCI score, lymph node metastases, and completeness of cytoreduction are important prognostic factors for survival in patients with primary peritoneal mesothelioma of epithelial origin [195, 196, 197, 198, 199].

##### 4.4.1.2 Role of IP Therapy

CRS combined with HIPEC improves mean survival by up to 4 years but with repeated procedures [194]. Data supporting the use of HIPEC following CRS in this disease has been largely based on retrospective studies demonstrating median OS ranging from 30 to 92 months using this approach [195, 197, 200, 201, 202, 203, 204, 205]. A multi‐institutional registry of retrospective data included 405 patients treated with CRS and HIPEC. The median survival was 53 months, and the 5‐year survival was 47% [201]. Another multi‐institutional study of 211 patients reported a median OS of 38 months with a 5‐year survival of 41% [202]. A meta‐analysis of 20 studies including 1047 patients reported a similar 5‐year survival of 42% in patients with complete or near complete cytoreduction prior to HIPEC [206]. In this study, the addition of EPIC and cisplatin‐based IP chemotherapy results in promising 5‐year survival rates [206].

Even in the absence of randomized data, CRS and IP therapy is considered first‐line therapy for primary peritoneal mesothelioma of epithelial origin. Perioperative systemic therapy should be considered for high‐risk features including Ki‐67 > 9%, nodal metastasis, PCI > 17, completeness of CCR > 1 or bicavitary disease. Outcomes are suboptimal for CRS and HIPEC in patients with sarcomatoid mesothelioma or biphasic disease. Surveillance in patients with a favorable prognostic profile such as CCR0, epithelioid subtype, no lymph node involvement, Ki‐67 ≤ 9%, or PCI ≤ 17 is reasonable as the benefit of adjuvant therapy is unknown in this population.

##### 4.4.1.3 Patterns of Recurrence Following CRS ± IP Therapy

Peritoneal recurrences are common following CRS and IP therapy for primary peritoneal mesothelioma [194].

#### 4.4.2 Subtopic 2/Hepatobiliary Cancers

PC from hepatobiliary cancers is relatively rare. Limited retrospective data report the use of CRS and HIPEC in this setting. A retrospective report of the use of CRS plus HIPEC in patients with hepatopancreaticobiliary malignancies with PC included nine patients with hepatocellular carcinoma, four with cholangiocarcinoma, three with gallbladder cancer, and one with pancreatic cancer. The PCI score, number of organs resected, completeness of cytoreduction, and 30‐day morbidity were equivalent. PFS was similar for all groups, but the median OS was longer in hepatocellular carcinoma (42 vs. cholangiocarcinoma 19 months, gallbladder 8 months, pancreatic 15 months, *p* = 0.206) resulting in improved 3‐year OS (59% hepatocellular carcinoma, vs. 0% for the other groups) [207].

Another small retrospective study specifically evaluated the use of CRS and HIPEC for the treatment of PC from gallbladder cancer after achieving complete cytoreduction (CCR0). Median and 3‐year OS were 22.4 months and 30%, respectively [208]. A larger retrospective study from China compared the prognosis of patients with intrahepatic cholangiocarcinoma with PC receiving CRS and HIPEC (51 patients) compared to CRS alone (61 patients). This study demonstrated a survival advantage associated with the use of HIPEC. The median OS was longer in the CRS + HIPEC group than in the CRS group (25.5 vs. 11.2 months, *p* < 0.001) [209].

Data supporting the use of CRS and HIPEC in patients with PC from hepatobiliary origin are scant. Anecdotal reports suggest that this approach is reasonable in carefully selected patients with limited disease. Further evidence is warranted.

#### 4.4.3 Subtopic 3/Other Metastatic Cancers

##### 4.4.3.1 Neuroendocrine Tumors

CRS and HIPEC may be considered as an appropriate treatment for the 20% of small‐bowel neuroendocrine tumors that present with PC. A retrospective study examined the role of CRS with or without HIPEC in patients with small bowel neuroendocrine tumors. The PCI and CCR scores were higher in the HIPEC group. HIPEC was associated with greater toxicity and a non‐significant trend toward improvement in PFS. No difference in OS was demonstrated. The study concluded that the addition of HIPEC to CRS did not provide additional benefit at the cost of increased toxicity [210].

##### 4.4.3.2 Other Metastatic Cancers

There is a lack of data supporting the use of CRS and IP therapy for PC associated with cancers originating outside of the abdominopelvic cavity.

### 4.5 TOPIC 5/Palliative Treatment Options for Patients Who Are Unsuitable or Decline Cytoreductive (Variant Case 7)

#### 4.5.1 Subtopic 1/Surgery With or Without IP Therapy

CRS and IP therapy may be a treatment option for advanced malignancies presenting with PC; the risk of morbidity may result in symptoms that impact quality of life. Not all patients with PC are considered candidates for CRS and IP therapy due to comorbid illness, and many cases of PC present with advanced, incurable disease not amenable to potentially curative treatments. Common symptoms associated with advanced PC include malignant bowel obstruction, fistulae, hydronephrosis, and symptomatic malignant ascites that can cause pain, nausea, anorexia, cachexia, and fatigue. For these cases, other therapies to manage complications and symptoms should be considered. Hereon, we describe therapies that offer effective palliative treatment in appropriately selected patients.

#### 4.5.2 Subtopic 2/Malignant Bowel Obstruction

Management of malignant bowel obstruction should include supportive care measures, such as fluid resuscitation for dehydration, nasogastric decompression for vomiting, and restriction of food and drink to minimize gastric stimulation and secretions. Imaging studies are helpful to determine the location and nature of the obstruction, and evaluation for perforation or ascites. Physical examination and evaluation of laboratory values, such as white blood count, can help assess for the presence of peritonitis. Observation is considered for patients who are hemodynamically stable without evidence of peritonitis to give the chance for obstruction to resolve without surgical intervention. Indications for urgent surgical intervention include patients with suspected peritonitis or imaging suggesting a closed loop obstruction. Palliative surgical interventions include resection of isolated obstruction, intestinal bypass, diverting ostomy, and/or placement of decompression gastrostomy tube. Because these procedures can be technically challenging and can be associated with a high risk of perioperative morbidity and mortality, careful patient selection is essential. Nonsurgical interventions for partial malignant bowel obstruction include low‐dose steroids to mitigate bowel wall edema and treat associated nausea, and somatostatin analogues to decrease secretions and stretching of the bowel wall that causes visceral pain.

#### 4.5.3 Subtopic 3/Fistulae

Digestive fistulae can occur spontaneously in patients with advanced PC or as enterocutaneous fistulae following CRS ± HIPEC. Fistulae can result in loss of electrolytes and fluids and can lead to a wide variety of pathophysiological complications including intraabdominal collection, wound infection, sepsis, malnutrition, and electrolyte imbalance. Treatment of fistulae usually involves somatostatin analogues, aiming to reduce volume output and total parenteral nutrition (TPN) primarily to prevent malnutrition and decrease the risk of infection. Spontaneous fistula closure has been reported to occur in 30%–40% [211].

#### 4.5.4 Subtopic 4/Hydronephrosis

Another common complication of PC is hydronephrosis from ureteral obstruction. Palliative treatments for hydronephrosis include ureteral stent placement, which is usually reserved for symptomatic patients or those with compromised renal function, or percutaneous nephrostomy tube if the ureter cannot be stented.

#### 4.5.5 Subtopic 5/Symptomatic Malignant Ascites

Paracentesis may provide immediate symptom relief for symptomatic ascites, although it is usually temporary. For patients requiring frequent paracentesis, a tunneled IP catheter that can be intermittently connected to a self‐contained vacuum drainage system may be considered. Other treatment options for the treatment of symptomatic ascites include percutaneous delivery of HIPEC (without CRS). Several studies have demonstrated the efficacy of HIPEC in the treatment of patients with malignant ascites [212, 213, 214, 215].

PIPAC is an emerging palliative treatment for patients with unresectable PC. Potential advantages of PIPAC over current treatment options are a homogeneous IP distribution, low local and systemic toxicity, and enhanced tumor penetration. Most of the early literature regarding the use of PIPAC in patients with unresectable PC is retrospective cohort or prospective phase I or II studies delivered in patients with PC from a variety of tumor origins used either as monotherapy or combined with systemic therapy and did not stratify results based on primary tumor, PIPAC ± systemic therapy, and line of palliative treatment. More recent studies supporting the use of PIPAC in the palliative setting focus on combined therapy using PIPAC with systemic treatments for specific tumor types [40, 216, 217, 218, 219, 220, 221, 222, 223, 224, 225, 226, 227].

#### 4.5.6 Subtopic 6/Palliative Systemic Therapy

Palliative systemic therapy is typically considered in patients with PC who cannot tolerate or decline aggressive local therapies. Choice of systemic therapies depends on the origin of the cancer, histology, and molecular profile (see Topic 1.). Systemic therapy can be given alone or combined with IP therapy.

## 5 Variant Cases

Variant cases were developed as examples for these guidelines to illustrate practical applications of consensus recommendations (Tables 2, 3, 4, 5, 6, 7, 8).

**TABLE 2:** Variant 1: Peritoneal carcinomatosis from ovarian cancer. 62‐year‐old female (BMI = 27; serum albumin 3.2 g/dL) with a good performance status (ECOG = 1) and newly diagnosed peritoneal carcinomatosis from ovarian primary. She presented with early satiety, abdominal distention, and obstipation. CA‐125 was elevated, CEA and CA 19‐9 were within normal limits, and contrast‐enhanced CT of the chest, abdomen, and pelvis demonstrated a 7 cm right complex ovarian mass, large volume ascites, omental caking, and no evidence of other metastatic disease. Laparoscopy confirmed findings on CT with miliary carcinomatosis in the pelvis, and laparoscopic PCI score was 12. Biopsy of peritoneal implant demonstrated high‐grade serous carcinoma with immunohistochemistry consistent with ovarian primary. She was evaluated by a multidisciplinary team and deemed fit for surgery.

Treatment	Rating category	Final tabulations									Group median rating	Disagree	SOE	SOR
		1	2	3	4	5	6	7	8	9				
*Treatment options*														
Cytoreductive surgery alone	U	0	8	5	2	1	0	1	0	0	3		—	↑
Neoadjuvant systemic therapy followed by interval cytoreductive surgery	A	0	0	0	1	1	2	5	4	2	7		S	↑
Primary cytoreductive surgery followed by adjuvant systemic therapy	A	0	0	0	1	1	2	4	6	1	7		M	↑
Intraperitoneal therapy														
Type of intraperitoneal therapy (if recommended)														
HIPEC^a^	A	0	0	0	0	2	2	10	1	0	7		M	↑
EPIC^a^	M	0	0	0	3	8	3	1	0	0	5		M	↑
Adjuvant intraperitoneal chemotherapy at the time of adjuvant systemic therapy	M	0	1	1	6	4	3	2	0	0	5		L	—
Systemic therapy alone		1	4	4	5	2	1	0	0	0	3	Y	EC	↑

*Note:* **Variant discussion**: ***In general***, the goal of aggressive treatment of PC of tubo‐ovarian origin is to achieve complete macroscopic resection (CCR0–1) followed by platinum‐based systemic therapy in patients who are deemed able to tolerate. In patients in whom initial optimal CRS is not feasible, neoadjuvant platinum‐based systemic therapy results in high rates of optimal CRS with similar results as initial surgery. For platinum‐resistant tumors, targeted therapy with PARP inhibitors may be considered. ***In this case***, consideration of IP therapy following interval CRS is warranted due to good performance status/fitness for surgery, and low laparoscopic PCI. Data most strongly supports the use of neoadjuvant chemotherapy followed by interval CRS and consideration of HIPEC or EPIC. Adjuvant IP chemotherapy can also be continued at the time of adjuvant systemic therapy. Systemic therapy alone would be considered a palliative option in this case. **Rating categories**: U (pink/red): usually not appropriate; M (yellow): may be appropriate; A (green): usually appropriate. **Disagree**: The variation of the individual ratings from the median rating indicates panel disagreement on the final recommendation. **SOE**: S: strong; M: moderate; L: limited; EC: expert consensus; EO: expert opinion. **SOR**: ↑: strong recommendation; ↓: weak recommendation; —: not strong, not weak.

**Abbreviations:** BMI: body mass index; CA 19‐9: carbohydrate antigen 19‐9; CA‐125: cancer antigen 125; CCR0–1: completeness of cytoreduction score with residual tumor deposits measuring no > 2 cm; CEA: carcinoembryonic antigen; CRS: cytoreductive surgery; CT: computed tomography; ECOG: Eastern Cooperative Oncology Group; EPIC: early postoperative intraperitoneal chemotherapy; HIPEC: hyperthermic intraperitoneal chemotherapy; IP: intraperitoneal; OS: overall survival; PARP: poly (ADP‐ribose) polymerase; PC: peritoneal carcinomatosis; PCI: Peritoneal Carcinomatosis Index; PFS: progression‐free survival.

^a^ IP therapy after interval CRS; IP therapy is not supported after upfront CRS.

**TABLE 3:** Variant 2: Primary peritoneal carcinomatosis (gynecologic origin). 70‐year‐old female with remote history of prior hysterectomy and bilateral salpingo‐oophorectomy for benign indications (BMI = 30; serum albumin 3.0 g/dL) with good performance status (ECOG = 1) and newly diagnosed primary peritoneal carcinomatosis. She presented with fatigue, abdominal distention, and anorexia. CA‐125 and CA 19‐9 were elevated, CEA was within normal limits, and contrast‐enhanced CT of the chest, abdomen, and pelvis demonstrated a large volume of ascites, omental caking, large right pleural effusion, and no evidence of other metastatic disease. Radiographic PCI score was 30. Image‐guided biopsy of one of the omental cakes demonstrated high‐grade serous carcinoma with inclusive immunohistochemistry. She was evaluated by a multidisciplinary team and was fit to undergo surgery.

Treatment	Rating category	Final tabulations									Group median rating	Disagree	SOE	SOR
		1	2	3	4	5	6	7	8	9				
*Treatment options*														
Cytoreductive surgery alone	U	5	5	3	1	1	0	0	0	0	2		M	↑
Neoadjuvant systemic therapy followed by consideration of interval cytoreductive surgery depending on response	A	0	0	0	0	0	2	6	4	3	7		S	↑
Primary cytoreductive surgery followed by adjuvant systemic therapy	M	1	0	2	2	5	4	0	0	1	5		M	↑
Intraperitoneal therapy														
Type of intraperitoneal therapy (if recommended)														
HIPEC^a^	A	0	0	0	2	2	1	11	1	0	7		M	↑
Adjuvant intraperitoneal chemotherapy at the time of adjuvant systemic therapy	M	0	0	0	3	10	4	0	0	0	5		L	—
Systemic therapy alone	M	0	2	1	6	5	2	0	1	0	4		EC	↑
Palliative therapy		0	3	3	7	4	0	0	0	0	4	Y	S	—

*Note:* **Variant discussion**: ***In general***, primary peritoneal carcinomatosis of serous histology is managed similarly to PC from serous ovarian carcinomas, with CRS combined with systemic chemotherapy and/or targeted therapy. ***In this case***, IP therapy should be cautiously considered in this patient with primary peritoneal carcinomatosis of gynecologic origin with large volume ascites, omental caking, large right pleural effusion and a high radiographic PCI. Even though the patient has a good performance status and is fit for surgery, the high PCI score indicates that maximal cytoreduction is unlikely. Neoadjuvant chemotherapy followed by reevaluation for interval CRS and consideration of HIPEC depending on response that would allow for optimal CRS is a reasonable approach. The likelihood that this chemo‐naïve patient will have a good response to neoadjuvant chemotherapy is relatively high. Palliative therapy should be considered for poor response to initial systemic therapy. **Rating categories:** U (pink/red): usually not appropriate; M (yellow): may be appropriate; A (green): usually appropriate. **Disagree**: The variation of the individual ratings from the median rating indicates panel disagreement on the final recommendation. **SOE**: S: strong; M: moderate; L: limited; EC: expert consensus; EO: expert opinion. **SOR**: ↑: strong recommendation; ↓: weak recommendation; —: not strong, not weak.

**Abbreviations**: BMI: body mass index; CA 19‐9: carbohydrate antigen 19‐9; CA‐125: cancer antigen 125; CEA: carcinoembryonic antigen; CRS: cytoreductive surgery; CT: computed tomography; ECOG: Eastern Cooperative Oncology Group; EPIC: early postoperative intraperitoneal chemotherapy; HIPEC: hyperthermic intraperitoneal chemotherapy; IP: intraperitoneal; PC: peritoneal carcinomatosis; PCI: Peritoneal Carcinomatosis Index.

^a^ IP therapy after interval CRS; IP therapy is not supported after upfront CRS.

**TABLE 4:** Variant 3: Peritoneal carcinomatosis from colorectal cancer. 45‐year‐old female (BMI = 30; serum albumin 3.0 g/dL) with good performance status (ECOG = 0) with a remote history of node‐positive right‐sided colorectal cancer treated with surgery and adjuvant chemotherapy presents with fatigue, abdominal tenderness, and anorexia. CEA was elevated, and CA‐125 and CA 19‐9 were within normal limits, and contrast‐enhanced CT of the chest, abdomen, and pelvis demonstrated < 10 peritoneal deposits located in the right upper quadrant of the abdomen measuring up to 8 mm and no evidence of nodal involvement or other metastatic disease. Radiographic PCI score was 13. Image‐guided biopsy of one of the peritoneal deposits confirmed adenocarcinoma consistent with colorectal primary. She was evaluated by a multidisciplinary team and deemed fit for surgery.

Treatment	Rating category	Final tabulations									Group median rating	Disagree	SOE	SOR
		1	2	3	4	5	6	7	8	9				
*Treatment options*														
Cytoreductive surgery alone	U	0	6	10	0	0	0	1	0	0	3		M	↑
Neoadjuvant systemic therapy followed by interval cytoreductive surgery	A	0	0	0	1	1	1	7	5	0	7		M	↑
Primary cytoreductive surgery followed by adjuvant systemic therapy		0	1	1	4	3	4	3	1	0	5	Y	M	—
Intraperitoneal therapy														
Type of intraperitoneal therapy (if recommended)														
HIPEC^a^	M	0	0	0	0	5	6	4	0	0	6		M	↑
EPIC^a^	M	0	0	0	1	9	4	1	0	0	5		M	↑
Systemic therapy alone	M	0	0	1	2	5	6	3	0	0	6		EC	↑

*Note:* **Variant discussion:** ***In general***, the addition of CRS to partial colectomy and perioperative systemic therapy can be considered in select patients with PC from CRC. Optimal candidates for consideration of CRS include extent of peritoneal involvement (ideally PCI score < 17), and patient performance status. Best outcomes are achieved in node‐negative patients with CCR0–1 CRS. Four randomized and numerous retrospective studies evaluating the efficacy of HIPEC following CRS for treatment of PC in patients with CRC have reported mixed results. Each of the studies have been criticized for various design and at present, the optimal IP chemotherapy regimen remains unclear. ***In this case***, CRS with or without IP therapy in combination with perioperative systemic therapy (neoadjuvant preferred) could be considered in this patient with peritoneal‐only progression due to good performance status/fitness for surgery, and low radiographic PCI. Systemic therapy alone would also be considered a reasonable option. **Rating categories:** U (pink/red): usually not appropriate; M (yellow): may be appropriate; A (green): usually appropriate. **Disagree**: The variation of the individual ratings from the median rating indicates panel disagreement on the final recommendation. **SOE**: S: strong; M: moderate; L: limited; EC: expert consensus; EO: expert opinion. **SOR**: ↑: strong recommendation; ↓: weak recommendation; —: not strong, not weak.

**Abbreviations:** BMI: body mass index; CA 19‐9: carbohydrate antigen 19‐9; CA‐125: cancer antigen 125; CCR0–1: completeness of cytoreduction score with residual tumor deposits measuring no > 2 cm; CEA: carcinoembryonic antigen; CRC: colorectal cancer; CRS: cytoreductive surgery; CT: computed tomography; ECOG: Eastern Cooperative Oncology Group; EPIC: early postoperative intraperitoneal chemotherapy; HIPEC: hyperthermic intraperitoneal chemotherapy; IP: intraperitoneal; PC: peritoneal carcinomatosis; PCI: Peritoneal Carcinomatosis Index.

^a^ Primary CRS ± IP therapy or interval CRS ± IP therapy following neoadjuvant systemic therapy can be considered. Neoadjuvant chemotherapy does not appear to increase perioperative morbidity or mortality.

## 6 Summary of Recommendations

1. The panel recommends that CRS followed by IP chemotherapy (HIPEC or EPIC) in combination with neoadjuvant and/or adjuvant systemic therapy may be appropriate for the typical case of PC from ovarian cancer origin that is able to undergo CCR0–1 cytoreduction [Variant 1].
2. The panel recommends that neoadjuvant chemotherapy followed by CRS (in patients with good response) and IP chemotherapy are usually appropriate for the typical case of PPC of gynecologic origin in a patient who is fit for surgery [Variant 2].
3. The panel recommends with reservations that CRS followed by IP therapy (HIPEC or EPIC) in combination with neoadjuvant/adjuvant chemotherapy may be appropriate for the typical case of PC from CRC origin that is able to undergo CCR0–1 cytoreduction [Variant 3].
4. The panel recommends that CRS followed by IP therapy (HIPEC or EPIC) is usually appropriate for the typical case of PC from low‐grade mucinous appendiceal origin in a patient that is fit for surgery. Systemic therapy is not recommended and is typically reserved for node‐positive disease [Variant 4].
5. The panel recommends with reservations that neoadjuvant chemotherapy followed by CRS (in patients without disease progression) and IP therapy may be appropriate for the typical case of limited PC from gastric cancer [Variant 5].
6. The panel recommends that CRS followed by IP therapy (HIPEC or EPIC) is usually appropriate for the typical case of primary peritoneal mesothelioma that is able to undergo CCR0–1 cytoreduction in a patient that is fit for surgery [Variant 6].
7. The panel recommends strongly that palliative therapies are usually appropriate for the typical case of PC in patients who are deemed able to tolerate but are unable to undergo optimal cytoreduction, or decline aggressive local therapy [Variant 7].

Table 9 provides a summary of evidence‐based recommendations for treatment of PC from different tumor origins.

**TABLE 5:** Variant 4: Peritoneal carcinomatosis from mucinous appendiceal neoplasm. 56‐year‐old male (BMI = 29; serum albumin 3.0 g/dL) with good performance status (ECOG = 1) and newly diagnosed appendiceal cancer with peritoneal carcinomatosis. He presented with abdominal pain and distention, and constipation. CEA was elevated, CA‐125 and CA 19‐9 were within normal limits, and contrast‐enhanced CT of the chest, abdomen, and pelvis demonstrated a large volume of ascites and omental caking, a 3 cm mass arising near the appendix, and no evidence of nodal or other metastatic disease. Radiographic PCI score was 15. Colonoscopy showed mucosal thickening at the appendiceal orifice and biopsy demonstrates low‐grade mucinous neoplasm. He was evaluated by a multidisciplinary team and deemed to be fit for surgery.

Treatment	Rating category	Final tabulations									Group median rating	Disagree	SOE	SOR
		1	2	3	4	5	6	7	8	9				
*Treatment options*														
Cytoreductive surgery ± IP therapy	A	0	0	0	0	3	1	7	3	1	7		S	↑
Neoadjuvant systemic therapy^a^ followed by interval cytoreductive surgery	U	2	7	6	0	0	0	0	0	0	2		M	↑
Primary cytoreductive surgery followed by adjuvant systemic therapy^a^	U	1	6	7	0	0	0	1	0	0	3		M	↑
Intraperitoneal therapy														
Type of intraperitoneal therapy (if recommended)														
HIPEC	M	0	0	0	0	2	9	2	2	0	6		S	↑
EPIC	M	0	0	0	3	9	1	2	0	0	5		S	↑

*Note:* **Variant discussion**: ***In general***, outcomes for patients with PC from appendiceal origin depend primarily on histology, extent of peritoneal involvement, completeness of cytoreduction, and patient performance status. Appendiceal tumors are typically grouped based on mucin production and in general, mucin‐producing appendiceal neoplasms are associated with a less aggressive biologic behavior than non‐mucinous tumors. Low‐grade mucinous tumors are associated with a better prognosis but do not appear to derive as much benefit from the addition of systemic therapy. The combination of CRS and IP therapy (HIPEC or EPIC) is considered the best treatment for patients with PC from appendiceal origin. The completeness of CRS (PCI < 19 predictive of CCR0) is the most important prognostic factor. Several retrospective series have reported encouraging results for HIPEC/EPIC in addition to CRS and the benefit appears to vary according to PCI, extent of cytoreduction, presence of nodal involvement, and tumor grade, and should be considered in patients who achieve CCR0 or CCR1 during CRS. There are no phase III data comparing EPIC with HIPEC. ***In this case***, consideration of CRS followed by IP therapy is warranted in this patient with PC from node‐negative, low‐grade mucinous appendiceal carcinoma with large volume ascites and omental caking, due to good performance status/fitness for surgery, and low radiographic PCI. Data supports maximal CRS and consideration of HIPEC or EPIC, especially if CCR0–1 is achieved. Systemic therapy is associated with limited benefit for patients with low grade mucinous appendiceal PC. **Rating categories**: U (pink/red): usually not appropriate; M (yellow): may be appropriate; A (green): usually appropriate. **Disagree**: The variation of the individual ratings from the median rating indicates panel disagreement on the final recommendation. **SOE**: S: strong; M: moderate; L: limited; EC: expert consensus; EO: expert opinion. **SOR**: ↑: strong recommendation; ↓: weak recommendation; —: not strong, not weak.

**Abbreviations:** BMI: body mass index; CA 19‐9: carbohydrate antigen 19‐9; CA‐125: cancer antigen 125; CCR0–1: completeness of cytoreduction score with residual tumor deposits measuring no > 2 cm; CEA: carcinoembryonic antigen; CRS: cytoreductive surgery; CT: computed tomography; ECOG: Eastern Cooperative Oncology Group; EPIC: early postoperative intraperitoneal chemotherapy; HIPEC: hyperthermic intraperitoneal chemotherapy; IP: intraperitoneal; PC: peritoneal carcinomatosis; PCI: Peritoneal Carcinomatosis Index.

^a^ Low‐grade mucinous tumors tend to have little to no benefit from the addition of systemic chemotherapy to IP therapy.

**TABLE 6:** Variant 5: Peritoneal carcinomatosis from gastric cancer. 55‐year‐old female (BMI = 22; serum albumin = 3.5 g/dL) with diffuse abdominal discomfort and 10 lb unintentional weight loss over 2 months, good performance status (ECOG = 1), and newly diagnosed poorly differentiated adenocarcinoma of the stomach with signet‐ring features is found to have four small biopsy‐proven, localized, perigastric peritoneal deposits (all < 5 mm) during staging laparoscopy. There is no evidence of ascites, and peritoneal washing cytology is otherwise negative. Contrast‐enhanced CT of the chest, abdomen, and pelvis demonstrates diffuse gastric wall thickening and a 1.5 cm suspicious gastro‐hepatic lymph node, and no evidence of other metastatic disease. She was evaluated by a multidisciplinary team and deemed to be fit for surgery.

Treatment	Rating category	Final tabulations									Group median rating	Disagree	SOE	SOR
		1	2	3	4	5	6	7	8	9				
*Treatment options*														
Cytoreductive surgery alone	U	0	6	6	2	0	1	0	0	0	3		L	↑
Systemic therapy with consideration of interval cytoreductive surgery depending on response^a^	A	0	0	0	0	2	1	8	4	0	7		M	↑
Primary cytoreductive surgery followed by adjuvant systemic therapy	U	0	6	6	1	2	0	2	0	0	3		L	↑
Intraperitoneal therapy														
Type of intraperitoneal therapy (if recommended)														
HIPEC^a^	M	0	0	1	3	5	4	2	0	0	5		M	↑
EPIC^a^	M	0	0	1	3	7	4	0	0	0	5		M	↑
Systemic therapy alone	A	0	0	0	2	1	1	8	3	0	7		EC	↑

*Note:* **Variant discussion:** ***In general***, PC from gastric cancer is associated with poor prognosis and median survival is approximately 6 months when treated with palliative chemotherapy alone. Retrospective and case–control studies demonstrate encouraging results for patients with PC from gastric cancer treated with CRS plus HIPEC in addition to systemic therapy, and may be considered in select patients with adequate performance status and are considered candidates for potential complete cytoreduction. The completeness of CRS is essential to achieve optimal outcomes. Despite encouraging results, CRS and IP therapy is not universally considered standard of care treatment regimen for gastric cancer patients with PC, owing largely to the absence of randomized clinical trials. Systemic therapy alone or other palliative measures is a reasonable consideration, especially in patients with poor performance status. ***In this case***, in a patient with intra‐abdominal‐only disease and low‐volume peritoneal implants it would be reasonable to consider neoadjuvant systemic therapy followed by interval CRS with consideration of HIPEC or EPIC depending on response, if the patient is able to undergo CRS and achieve CCR0–1. Systemic therapy alone would be considered a reasonable option. **Rating categories**: U (pink/red): usually not appropriate; M (yellow): may be appropriate; A (green): usually appropriate. **Disagree**: The variation of the individual ratings from the median rating indicates panel disagreement on the final recommendation. **SOE**: S: strong; M: moderate; L: limited; EC: expert consensus; EO: expert opinion. **SOR**: ↑: strong recommendation; ↓: weak recommendation; —: not strong, not weak.

**Abbreviations:** BMI: body mass index; CCR0–1: completeness of cytoreduction score with residual tumor deposits measuring no > 2 cm; CRS: cytoreductive surgery; CT: computed tomography; ECOG: Eastern Cooperative Oncology Group; EPIC: early postoperative intraperitoneal chemotherapy; HIPEC: hyperthermic intraperitoneal chemotherapy; IP: intraperitoneal; PC: peritoneal carcinomatosis.

^a^ Interval CRS + IP therapy following neoadjuvant systemic therapy can be considered depending on response and patient performance status.

**TABLE 7:** Variant 6: Primary peritoneal mesothelioma. 55 year‐old male who undergoes surgery for an umbilical hernia (BMI = 24; serum albumin 3.8 g/dL) with good performance status (ECOG = 1) who is found to have nodules in the excised hernia sac, and pathology is consistent with epithelioid malignant peritoneal mesothelioma. Contrast‐enhanced CT of the chest, abdomen, and pelvis demonstrated a diffuse thickening of the omentum with scattered calcifications, perihepatic soft tissue thickening, and no evidence of nodal or other metastatic disease. Radiographic PCI score was 17. He was evaluated by a multidisciplinary team and deemed to be fit for surgery.

Treatment	Rating category	Final tabulations									Group median rating	Disagree	SOE	SOR
		1	2	3	4	5	6	7	8	9				
*Treatment options*														
Cytoreductive surgery ± IP therapy	A	0	0	0	0	1	3	10	1	1	7		M	↑
Neoadjuvant systemic therapy followed by interval cytoreductive surgery ± IP therapy	M	1	0	3	8	2	1	0	0	0	4		L	↑
Primary cytoreductive surgery ± IP therapy followed by adjuvant systemic therapy	M	0	0	2	7	3	2	1	0	0	4		S	↑
Intraperitoneal therapy														
Type of intraperitoneal therapy (if recommended)														
HIPEC	A	0	0	1	0	1	3	12	0	0	7		S	↑
EPIC	M	0	0	0	2	10	2	1	0	0	5		S	↑
Systemic therapy alone	M	0	0	1	7	2	4	1	0	0	4		EC	↑

*Note:* **Variant discussion**: ***In general***, primary peritoneal mesothelioma is a rare tumor of peritoneal origin which accounts for approximately 30% of all mesotheliomas, and is usually diagnosed at an advanced stage with widespread disease throughout the peritoneum. Since diffuse abdominal peritoneal involvement characterizes this disease, aggressive local therapy with optimal cytoreduction with or without IP therapy is a reasonable approach in patients who can tolerate the procedure. Data supporting the use of HIPEC, EPIC and cisplatin‐based IP chemotherapy results in promising 5‐year survival rates. Even in the absence of randomized data CRS and IP therapy is considered first‐line therapy for primary peritoneal mesothelioma in patients who can tolerate the therapy. Perioperative systemic therapy should be considered for high‐risk features including Ki‐67 > 9%, nodal metastasis, PCI > 17, completeness of CCR > 1, biphasic disease, or bicavitary disease. Surveillance in patients with favorable prognostic profile such as CCR0, epithelioid subtype, no lymph node involvement, Ki‐67 ≤ 9%, or PCI ≤ 17 is reasonable as the benefit of adjuvant therapy is unknown in this population. Consideration of palliative treatment options should be reserved for patients who cannot tolerate therapy, decline aggressive surgery are refractory to therapy, or in whom maximal cytoreduction is not achievable. ***In this case***, CRS and IP therapy is considered first‐line therapy in this patient with primary epithelial peritoneal mesothelioma with a good performance status and is fit for surgery, and with low volume peritoneal‐only disease. Data supports maximal CRS with HIPEC or EPIC, especially if CCR0–1 is achieved. **Rating categories:** U (pink/red): usually not appropriate; M (yellow): may be appropriate; A (green): usually appropriate. **Disagree**: The variation of the individual ratings from the median rating indicates panel disagreement on the final recommendation. **SOE**: S: strong; M: moderate; L: limited; EC: expert consensus; EO: expert opinion. **SOR**: ↑: strong recommendation; ↓: weak recommendation; —: not strong, not weak.

**Abbreviations:** BMI: body mass index; CCR > 1: completeness of cytoreduction score with residual tumor deposits measuring > 2 cm; CRS: cytoreductive surgery; CT: computed tomography; ECOG: Eastern Cooperative Oncology Group; EPIC: early postoperative intraperitoneal chemotherapy; HIPEC: hyperthermic intraperitoneal chemotherapy; IP: intraperitoneal; PCI: Peritoneal Carcinomatosis Index.

**TABLE 8:** Variant 7: Peritoneal carcinomatosis from colorectal cancer (palliative). 64‐year‐old cachectic female with a remote history of colon cancer, poor performance status (ECOG = 2), uncontrolled HTN, and COPD on 2 L supplemental O_2_ presented with an unexpected weight loss of 20 lbs over the last 6 weeks (BMI = 17; serum albumin 2.2 g/dL), with anorexia and fatigue. On exam, there was abdominal distention and a subtle, nontender mass palpated in the right lower quadrant of the abdomen. The remainder of the exam was non‐focal. Contrast‐enhanced CT scan of the chest, abdomen, and pelvis revealed peritoneal carcinomatosis most heavily concentrated in the right lower quadrant associated with a moderate amount of ascites, no evidence of bowel obstruction, and no evidence of other metastatic disease. Diagnostic laparoscopy confirmed bulky peritoneal implants measuring up to 4 cm and diffuse omental caking with a PCI score estimated at 32. Biopsy of the peritoneal implants revealed high‐grade adenocarcinoma with immunohistochemistry consistent with colorectal primary. She was evaluated by a multidisciplinary team and deemed to be unfit for surgery.

Treatment	Rating category	Final tabulations									Group median rating	Disagree	SOE	SOR
		1	2	3	4	5	6	7	8	9				
*Treatment options*														
Cytoreductive surgery ± intraperitoneal therapy	U	3	6	4	2	0	0	0	0	0	2		S	↑
Palliative therapy														
Type of palliative therapy (if recommended)														
Paracentesis	A	0	0	0	0	2	0	8	4	1	7		EC	↑
Cytoreductive surgery	U	4	7	3	1	0	0	0	0	0	2		M	↑
HIPEC		0	2	4	1	8	1	1	0	0	5	Y	M	—
PIPAC	M	0	1	2	2	9	1	2	0	0	5		M	↑
Systemic therapy		0	0	0	0	4	4	8	1	0	7		EC	↑
Supportive care	A	0	0	0	0	1	1	7	2	4	7		EC	↑

*Note:* **Variant discussion**: ***In general***, not all patients with PC are considered candidates for CRS and IP therapy due to comorbid illness, and many cases of PC present with advanced, incurable disease not amenable to potentially curative treatments. Common symptoms associated with advanced PC include malignant bowel obstruction, fistulae, hydronephrosis, and symptomatic malignant ascites that can cause pain, nausea, anorexia, cachexia, and fatigue. For these cases, other therapies to manage symptoms should be considered. In patients with symptomatic malignant ascites, paracentesis may provide immediate symptom relief but is usually temporary. For patients requiring frequent paracentesis, a tunneled IP catheter can be intermittently connected to a self‐contained vacuum drainage system. Other treatment options for treatment of symptomatic ascites include percutaneous delivery of HIPEC (without CRS). PIPAC is an emerging palliative treatment for patients with unresectable PC. Potential advantages of PIPAC are a homogeneous IP distribution, low local and systemic toxicity, and enhanced tumor penetration. Palliative systemic therapy is typically considered in patients with PC who cannot tolerate or decline aggressive local therapies. Ultimately, choice of palliative therapy depends largely on patient performance status, extent of disease, and preference, with supportive care as a reasonable option for patients who cannot tolerate or decline other palliative measures. ***In this case***, CRS should not be considered in this patient with symptomatic PC who is unfit for surgery. Paracentesis can be considered for immediate symptom relief. HIPEC or PIPAC can be considered for more durable palliation in patients deemed able to tolerate. Systemic therapy should also be considered, and other supportive measures should be incorporated in the care of this patient. **Rating categories**: U (pink/red): usually not appropriate; M (yellow): may be appropriate; A (green): usually appropriate. **Disagree**: The variation of the individual ratings from the median rating indicates panel disagreement on the final recommendation. **SOE**: S: strong; M: moderate; L: limited; EC: expert consensus; EO: expert opinion. **SOR**: ↑: strong recommendation; ↓: weak recommendation; —: not strong, not weak.

**Abbreviations**: BMI: body mass index; COPD: chronic obstructive pulmonary disease; CRS: cytoreductive surgery; CT: computed tomography; ECOG: Eastern Cooperative Oncology Group; HIPEC: hyperthermic intraperitoneal chemotherapy; HTN: hypertension; IP: intraperitoneal; PC: peritoneal carcinomatosis; PCI: Peritoneal Carcinomatosis Index; PIPAC: pressurized intraperitoneal aerosolized chemotherapy.

**TABLE 9:** Summary of evidence‐based recommendations for peritoneal carcinomatosis from different tumor origins.

Case scenario	Treatment		Variant discussion
	Usually appropriate	May be appropriate	
PC from ovarian cancer Newly diagnosed, 7 cm right complex ovarian mass, large volume ascites, miliary implants, omental caking, and no evidence of other metastatic disease. High‐grade serous carcinoma Laparoscopic PCI = 12 Fit for surgery	‐Neoadjuvant systemic therapy followed by interval CRS + IP therapy (HIPEC or EPIC [1]) ‐Primary CRS followed by adjuvant systemic therapy	‐Adjuvant IP chemotherapy at the time of adjuvant systemic therapy	*Peritoneal carcinomatosis of ovarian origin is managed with CRS combined with systemic chemotherapy and/or targeted therapy. Platinum‐based systemic therapy is typically recommended in the neoadjuvant or adjuvant setting, with PARP inhibitors combined with chemotherapy for platinum‐resistant tumors *The goal is maximal cytoreduction resulting in residual tumor deposits measuring no > 2 cm (CCR0–1) *Consideration of peritoneal therapy is warranted in this patient with high grade ovarian serous carcinoma with miliary peritoneal implants and ascites due to good performance status/fitness for surgery, and low laparoscopic PCI *High quality data is lacking to support the use of IP therapy at the time of primary debulking. Data most strongly supports the use of neoadjuvant chemotherapy followed by interval CRS and consideration of HIPEC or EPIC *Adjuvant IP chemotherapy can also be continued at the time of adjuvant systemic therapy
Primary PC from gynecologic cancer Remote history of TAH BSO for benign indications Newly diagnosed, large volume ascites, omental caking, large right pleural effusion, and no evidence of other metastatic disease. High‐grade serous carcinoma c/w gynecologic origin Radiographic PCI = 30 Fit for surgery	‐Neoadjuvant systemic therapy followed by interval CRS + IP therapy (HIPEC or EPIC [1])	‐Primary CRS followed by adjuvant systemic therapy + IP therapy (HIPEC or EPIC [1]) ‐Systemic therapy alone	*Primary peritoneal carcinomatosis of serous histology is managed similarly to PC from serous ovarian carcinomas *In this case, IP therapy should be cautiously considered in this patient with primary peritoneal carcinomatosis of gynecologic origin with large volume ascites, omental caking, large right pleural effusion and a high radiographic PCI, indicating that maximal cytoreduction is unlikely *Neoadjuvant chemotherapy followed by reevaluation for interval CRS and consideration of HIPEC depending on response that would allow for optimal CRS is a reasonable approach. The likelihood that this chemo‐naïve patient will have a good response to neoadjuvant chemotherapy is relatively high *Palliative therapy should be considered for poor response to initial systemic therapy
PC from CRC Remote history of node‐positive, right‐sided CRC Surveillance imaging shows < 10 peritoneal deposits located in the right upper quadrant, all < 8 mm, no evidence of nodal involvement or other metastatic disease. Biopsy‐proven adenocarcinoma c/w CRC origin Radiographic PCI = 13 Fit for surgery	‐Neoadjuvant systemic therapy followed by interval CRS + IP therapy (HIPEC or EPIC [1])	‐Primary CRS followed by adjuvant systemic therapy + IP therapy (HIPEC or EPIC [1]) ‐Systemic therapy alone	*The addition of CRS to partial colectomy to remove the tumor can be considered in select patients with PC from CRC and optimal candidates for consideration of CRS include extent of peritoneal involvement (ideally PCI score < 17), and patient performance status *Four randomized and numerous retrospective studies evaluating the efficacy of HIPEC following CRS for treatment of PC in patients with CRC have reported mixed results. Each of the studies have been criticized for various design limitations but it appears that the benefit of CRS and HIPEC in addition to systemic therapy compared to systemic therapy alone is greater for those patients with PC only (compared with those with other involved sites) with low PCI in whom CCR‐0 is achievable *In this case, for peritoneal only recurrence of CRC, CRS followed by IP therapy in combination with neoadjuvant/adjuvant systemic therapy could be considered in this patient with peritoneal‐only progression of a previously treated CRC with low‐volume peritoneal implants due to good performance status/fitness for surgery, and low radiographic PCI *Systemic therapy alone would also be considered a reasonable option
PC from low‐grade mucinous appendiceal neoplasm Newly diagnosed, large volume ascites and omental caking, 3 cm mass arising near the appendix, and no evidence of nodal or other metastatic disease. Colonoscopy showed mucosal thickening at the appendiceal orifice Low grade mucinous neoplasm Radiographic PCI = 15 Fit for surgery	‐Cytoreductive surgery ± IP therapy	‐Use of HIPEC or EPIC [1]	*Outcomes for patients with PC from appendiceal origin depend primarily on histology, extent of peritoneal involvement, completeness of cytoreduction, and patient performance status *Appendiceal tumors are typically grouped based on mucin production and in general, mucin‐producing appendiceal neoplasms are associated with a less aggressive biologic behavior than non‐mucinous tumors *Low‐grade mucinous tumors are associated with a better prognosis but do not appear to derive as much benefit from the addition of systemic therapy to CRS and IP therapy compared to higher‐grade tumors *The combination of CRS and IP therapy (HIPEC or EPIC) is considered the best treatment for patients with PC from appendiceal origin, even in the setting of incomplete cytoreduction where patients can still experience improved survival and symptom control *The completeness of CRS is the most important prognostic factor for patients with PC from appendiceal origin and PCI < 19 appears to be predictive of CCR0
PC from gastric cancer Newly diagnosed gastric adenocarcinoma with diffuse gastric wall thickening and a 1.5 cm suspicious gastro‐hepatic lymph node, and no evidence of other metastatic disease. Staging laparoscopy demonstrates four small biopsy‐proven, localized, perigastric peritoneal deposits (all < 5 mm) Poorly differentiated adenocarcinoma of the stomach with signet‐ring features Fit for surgery	‐Systemic therapy with consideration of interval CRS depending on response ‐Systemic therapy alone	‐Use of HIPEC or EPIC [1]	*PC associated with gastric cancer is associated with a poor prognosis and CRS with or without IP therapy is not accepted as standard of care even though promising data supports this approach in patients who are able to undergo CRS and achieve CCR0–1 *Retrospective and case–control studies demonstrate encouraging results for patients with PC from gastric cancer treated with CRS plus HIPEC in addition to systemic therapy and may be considered in select patients with adequate performance status and are considered candidates for potential complete cytoreduction, depending on response to neoadjuvant therapy and performance status *Due to good performance status in this patient with intra‐abdominal‐only disease and low‐volume peritoneal implants, it would be reasonable to cautiously consider neoadjuvant systemic therapy followed by interval CRS with consideration of HIPEC or EPIC, depending on response. While there is increasing interest in exploring the use neoadjuvant IP chemotherapy and systemic therapy with the goal of optimizing cytoreduction, there is no high‐level evidence to suggest improvements in outcomes using this approach *Because PC from gastric cancer is associated with poor prognosis, systemic therapy alone or other palliative measures are reasonable considerations, especially in patients with poor performance status
Primary peritoneal mesothelioma Incidental finding following surgery for umbilical hernia, with nodules found in the excised hernia sac. Imaging identified diffuse thickening of the omentum with scattered calcifications, perihepatic soft tissue thickening, and no evidence of nodal or other metastatic disease Epithelioid malignant peritoneal mesothelioma Radiographic PCI = 17 Fit for surgery	‐Cytoreductive surgery ± IP therapy (HIPEC preferred) ‐Neoadjuvant systemic therapy followed by interval CRS ± IP therapy ‐Primary CRS ± IP therapy followed by adjuvant systemic therapy	‐Use of EPIC [1] ‐Systemic therapy alone	*Primary peritoneal mesothelioma is a rare tumor of peritoneal origin which accounts for approximately 30% of all mesotheliomas and is usually diagnosed at an advanced stage with widespread disease throughout the peritoneum *The best treatment option for a medically operable patient in whom cytoreduction is includes CRS and IP therapy (HIPEC or EPIC). Even with incomplete cytoreduction, patients can experience improved survival and symptomatic control *Data supporting the use of HIPEC following CRS in this disease has been largely based on retrospective studies and demonstrates improvement in survival compared to palliative therapies *Perioperative systemic therapy should be considered for high‐risk features including Ki‐67 > 9%, nodal metastasis, PCI > 17, completeness of CCR > 1, biphasic disease, or bicavitary disease *Surveillance in patients with favorable prognostic profile such as CCR0, epithelioid subtype, no lymph node involvement, Ki‐67 ≤ 9%, or PCI ≤ 17 is reasonable as the benefit of adjuvant therapy is unknown in this population

*Note:* Colored indicates only for ease of reading to separate the different cases.

## Author Contributions

**Timothy Kennedy:** writing – original draft (supporting), writing – review and editing (lead). **Sareena Singh:** writing – original draft (supporting), writing – review and editing (lead). **Gerard Abood:** writing – original draft (supporting), writing – review and editing (lead). **Eric Christenson:** writing – original draft (supporting), writing – review and editing (lead). **Christopher J. Anker:** methodology (equal), writing – review and editing (supporting). **Dmitriy Akselrod:** writing – review and editing (supporting). **Christopher L. Hallemeier:** writing – review and editing (supporting). **Krishan R. Jethwa:** writing – review and editing (supporting). **Ed Kim:** writing – review and editing (supporting). **Percy Lee:** writing – review and editing (supporting). **Eric D. Miller:** writing – review and editing (supporting). **Neil B. Newman:** writing – review and editing (supporting). **J. Eva Selfridge:** writing – review and editing (supporting). **Navesh Sharma:** writing – review and editing (supporting). **William Small Jr:** writing – review and editing (supporting). **Leila Tchelebi:** writing – review and editing (supporting). **Vonetta M. Williams:** writing – review and editing (supporting). **Suzanne Russo:** conceptualization (equal), methodology (equal), writing – original draft (lead), writing – review and editing (lead).

## Ethics Statement

The authors have nothing to report.

## Consent

The authors have nothing to report.

## Conflicts of Interest

All panelists were required to declare all conflicts of interest for the previous 36 months prior to initiating work on this document. These complete disclosure forms are retained by the ARS in perpetuity. The ARS AUC Steering Committee reviewed these disclosures with the chair and co‐chair of this document and approved the participation of the panelists prior to starting the development of this work. Disclosures potentially relevant to the content of this guideline are provided: ^4^Dr. Christensen reports grants from Haystack Oncology, Pfizer Inc., Affimed gmbh, and Nextcure; and consulting fees from Seres Therapeutics, SIRTEX. ^6^Dr. Akselrod reports consulting fees from Otsuka America Pharmaceutical. ^7^Dr. Jethwa reports honoraria from RadOncQuestions.com LLC. ^9^Dr. Lee reports honoraria from Varian, ViewRay, AstraZeneca; consulting fees from Varian, ViewRay, AstraZeneca, Genentech, Johnson & Johnson, Roche, RTOG foundation, and meeting and travel support from Radiosurgery Society. ^12^Dr. Selfridge reports drug support for clinical trials from Medpacto Inc. and Clinigen Inc. and industry‐sponsored studies from BioAtla, Arcus Biosciences, AVEO, Cue Biopharma, and Pfizer. ^14^Dr. Small reports personal fees from Carl Zeissand meeting and travel support for ACR, NRG Oncology, and Carl Zeiss.

## Data Availability

Data sharing is not applicable to this article as no new data were created or analyzed in this study.
